# Identification of distinct slow mode of reversible adaptation of pancreatic ductal adenocarcinoma to the prolonged acidic pH microenvironment

**DOI:** 10.1186/s13046-022-02329-x

**Published:** 2022-04-11

**Authors:** Tzu-Chin Wu, Chien-Yu Liao, Wei-Chien Lu, Chuang-Rung Chang, Fang-Yu Tsai, Shih-Sheng Jiang, Tsung-Hsien Chen, Kurt Ming-Chao Lin, Li-Tzong Chen, Wun-Shaing Wayne Chang

**Affiliations:** 1 National Institute of Cancer Research, National Health Research Institutes, Zhunan, 350401 Taiwan; 2 Institute of Molecular and Cellular Biology, College of Life Science, National Tsing Hua University, Hsinchu, 300044 Taiwan; 3 Institute of Biomedical Engineering and Nanomedicine, National Health Research Institutes, Zhunan, 350401 Taiwan; 4 Current address: Ditmanson Medical Foundation, Chia-Yi Christian Hospital, Chia-Yi, 60002 Taiwan

**Keywords:** Pancreatic ductal adenocarcinoma, Tumor microenvironment, Extracellular pH, Acidic stress, Mitochondrial dynamics, Fusion, Fission, Autophagy

## Abstract

**Background:**

Pancreatic ductal adenocarcinoma (PDAC) is the most common pancreatic neoplasm with high metastatic potential and poor clinical outcome. Like other solid tumors, PDAC in the early stages is often asymptomatic, and grows very slowly under a distinct acidic pH*e* (extracellular pH) microenvironment. However, most previous studies have only reported the fate of cancerous cells upon cursory exposure to acidic pH*e* conditions. Little is known about how solid tumors—such as the lethal PDAC originating within the pancreatic duct-acinar system that secretes alkaline fluids—evolve to withstand and adapt to the prolonged acidotic microenvironmental stress.

**Methods:**

Representative PDAC cells were exposed to various biologically relevant periods of extracellular acidity. The time effects of acidic pH*e* stress were determined with respect to tumor cell proliferation, phenotypic regulation, autophagic control, metabolic plasticity, mitochondrial network dynamics, and metastatic potentials.

**Results:**

Unlike previous short-term analyses, we found that the acidosis-mediated autophagy occurred mainly as an early stress response but not for later adaptation to microenvironmental acidification. Rather, PDAC cells use a distinct and lengthy process of reversible adaptive plasticity centered on the early fast and later slow mitochondrial network dynamics and metabolic adjustment. This regulates their acute responses and chronic adaptations to the acidic pH*e* microenvironment. A more malignant state with increased migratory and invasive potentials in long-term acidosis-adapted PDAC cells was obtained with key regulatory molecules being closely related to overall patient survival. Finally, the identification of 34 acidic pH*e*-related genes could be potential targets for the development of diagnosis and treatment against PDAC.

**Conclusions:**

Our study offers a novel mechanism of early rapid response and late reversible adaptation of PDAC cells to the stress of extracellular acidosis. The presence of this distinctive yet slow mode of machinery fills an important knowledge gap in how solid tumor cells sense, respond, reprogram, and ultimately adapt to the persistent microenvironmental acidification.

**Supplementary Information:**

The online version contains supplementary material available at 10.1186/s13046-022-02329-x.

## Background

Pancreatic ductal adenocarcinoma (PDAC), often simply described as pancreatic cancer, is the most prevalent neoplastic disease of the pancreas [1]. PDAC has a devastating 5-year survival rate of less than 10% [2], and is projected to soon become the second- or third-leading cause of cancer mortality in many developed countries [3, 4]. Like other solid tumors, PDAC adopts fermentative glycolysis as an additional metabolic mechanism to compensate for the impaired oxidative phosphorylation to support cell survival and proliferation [5, 6]. The elevated glucose consumption—coupled with increased excretion of lactate and protons from glycolytic cells to the poorly perfused extracellular spaces—leads to an acidification of the tumor microenvironment [7, 8].

Extracellular microenvironmental acidification is a common hallmark of solid tumors [8, 9]. Although still not fully understood, an acidic microenvironment with an inverted pH gradient of extracellular pH (pH*e*) lower than intracellular pH (pH*i*) appears to impose selective pressures whereby tumor cells must adapt or die [10, 11]. It is now assumed that acidic pH*e* modulates multiple functions of tumor cells including activation of protective autophagy [12–15], acquisition of anoikis resistance [15, 16], induction of epithelial to mesenchymal transition (EMT) [17, 18], promotion of local invasion [19], and enhancement of chemoresistance [20]. Hence, a better understanding of the impact of interstitial acidity and how tumor cells coordinate various adaptation mechanisms to cope with the acidified microenvironment is critical to improving therapeutic efficacy against solid cancers especially lethal PDAC.

Many solid tumors grow slowly, with an estimated average TVDT (tumor volume doubling time) of several months to multiple years [21–26]. Given the long latency of tumor development especially for initial primary lesions, the acidification of the extracellular microenvironment is generally considered to be a lengthy and sustained process. However, most literatures to date have examined solid tumor cells exposed to acidic pH*e* for a few minutes or hours up to days or weeks [13–18, 27, 28]. In our opinion, these short-term exposure experiments can only provide limited insights toward the early responses of tumor cells to extracellular acidotic stress. Little is known about how solid tumors, such as PDAC originated within the pancreatic duct-acinar system that secretes alkaline fluids, evolve to withstand and adapt to the prolonged acidic microenvironment and then progress into more advanced stages.

Here, we established and characterized PDAC tumor cells exposed to different periods of acidic pH*e* stress. Unlike previous short-term studies, we show that acidosis-mediated autophagy occurred mainly as an early stress response but not in later adaptation to the prolonged extracellular acidification. Rather, PDAC cells employ a distinct long-term process of reversible adaptive plasticity, centering on the early fast and later slow mitochondrial network dynamics and metabolic reprogramming, for acute responses and chronic adaptations to the acidic pH*e* microenvironment. A continued advance toward more aggressive phenotypic states in PDAC cells under extended extracellular acidity was observed, with several major gene clusters functionally related to the long-term acidic pH*e* exposures. Finally, 34 potential target genes were found significantly associated with the overall survival of cancer patients—most of these were previously unable to be identified by short-term analyses of the effects of microenvironmental acidification on solid tumors.

## Methods

### Materials

List of antibodies was provided in Supplementary Table S1. Unless otherwise specified, all reagents and chemicals were purchased from either Thermo Fisher Scientific (Carlsbad, CA, USA) or Sigma-Aldrich by Merck (Darmstadt, Germany).

### Cell lines and cultures

Human pancreatic ductal adenocarcinoma SUIT-2 and BxPC-3 cell lines were freshly acquired from JCRB (Japanese Collection of Research Bioresources, National Institutes of Biomedical Innovation, Health and Nutrition, Osaka, Japan) and BCRC (Bioresource Collection and Research Center, Hsinchu, Taiwan) cell banks, respectively. The STR (short tandem repeat) profiles of both cell lines were validated by FIRDI (Food Industry Research and Development Institute, Hsinchu, Taiwan), and the certificates of authentication can be provided upon request. Cells were cultured as monolayers in RPMI-1640 medium (Cat. #31800-022, Gibco by Thermo Fisher Scientific, Waltham, MA, USA) supplemented with 23.8 mM NaHCO_3_ and 10% (v/v) FBS at 37 °C in a humidified atmosphere of 5% CO_2_ in air, and were routinely examined for mycoplasma contamination using the EZ-PCR mycoplasma detection kit (Biological Industries, Kibbutz Beit HaEmek, Israel). To assess the impact of acidic pH*e* on PDAC cells, the bicarbonated RPMI-1640 medium was further supplemented with 25 mM HEPES/PIPES (bioWORLD by GeneLinx, Dublin, OH, USA) followed by the addition of appropriate amount of 1 N HCl to either pH*e* 7.4 for use as the buffer control medium, or to pH*e* 6.7 for use as the acidic culture medium as per the guidelines recommended by Michl et al. [29]. Each cell sample was adapted to the intended pH by adding the corresponding pH-adjusted medium to freshly split cells, followed by intensive daily monitoring (e.g., before and after each experiment or when cultivation took place in the humidified CO_2_ incubator) to ensure the desired medium pH level to be accurate within 0.1 units throughout experiments. To achieve statistical power, all cell samples subjected to different periods of acidic exposures were prepared in 3 separate experiments.

### Cell viability and proliferation assays

Cell viability was determined by trypan blue (Invitrogen by Thermo Fisher Scientific, Eugene, OR, USA) dye exclusion assay and expressed as the percentage of living cells counted. To measure changes in cellular proliferation, the MTT (thiazolyl blue tetrazolium bromide) assay was performed. The blue formazan crystals were solubilized with DMSO, and the colored solution was read at 570 nm using a SpectraMax 250 microplate reader (Marshall Scientific, Hampton, NH, USA).

### Cell metabolic analysis

The oxygen consumption rate (OCR) and the extracellular acidification rate (ECAR) of test cell samples were assessed using a Seahorse XFe24 Extracellular Flux Analyzer (Agilent, Santa Clara, CA, USA) as per the guideline provided by the manufacture. Briefly, cells were seeded in the XFe24 microplates and cultured in conditioned medium for 16 h (37 °C under 5% CO_2_ and 95% humidity) to allow attachment and growth. Prior to the start of Seahorse assays, the optimal FCCP (carbonyl cyanide-4(trifluoromethoxy)phenylhydrazone) concentrations were determined by titration studies. For the OCR measurements, cells were washed in pre-warmed medium (2% FBS in RPMI-1640 without NaHCO_3_, pH adjusted to 7.4) and equilibrated at 37 °C for 1 h before the assay. For the ECAR measurements, cells were washed in pre-warmed glucose-free RPMI-1640 medium (Cat. R1383, Sigma-Aldrich by Merck) supplemented with 2% (v/v) FBS without NaHCO_3_, pH adjusted to 7.4 and equilibrated at 37 °C for 30 min before the experiments. Data were normalized by cell number, and graphs were calculated and plotted using Agilent Seahorse Wave Desktop software.

### Visualization of cellular morphology and mitochondrial cristae ultrastructure

To visualize cellular morphological changes, all cells cultured in their corresponding pH*e* medium were collected and plated in 35-mm diameter μ-dishes (Ibidi, Martinsried, Germany). A minimum of 10 randomly chosen microscopic fields (~ 70 cells per field) were imaged using an AF6000 LX microscope (Leica Microsystems, Singapore) equipped with Zyla sCMOS camera (Andor by Oxford Instruments, Belfast, UK) and objective lens (HCX PL Fluotar L 20x/0.40 CORR PH1) with 1.8x digital zoom. To observe mitochondrial morphology and cristae ultrastructure, the TEM (transmission electron microscopy) images of each cell group were taken on an H-7650 electron microscope (Hitachi High-Technologies, Tokyo, Japan) equipped with a CCD (charge-coupled device) camera (Advanced Microscopy Techniques, Danvers, MA, USA). The density of cristae was calculated based on 45 randomly selected mitochondria per cell group with ImageJ software (https://imagej.nih.gov/ij).

### Immunofluorescence analysis

To detect the changes of autophagy, approximately 2 × 10^4^ cells obtained from each sample were incubated with 200 nM LysoTracker Red DND-99 for lysosome visualization, followed by labeling with 1x CYTO-ID Green dye (Enzo Life Sciences, Farmingdale, NY, USA) for tracking autophagic vacuoles, and with 1 μg/mL Hoechst 33342 for nucleus staining. After incubation, the stained cells were subjected to image analysis by a TCS SP5 confocal microscope (Leica Microsystems). For ROS measurement, cells were stained with 5 μM CellROX Green dye for oxidative stress detection and then counterstained with 1 μg/mL Hoechst 33342 for confocal imaging. To determine mitochondrial superoxide production and membrane potential (∆ψ mt), cells were respectively stained with 5 μM MitoSOX Red and 200 nM TMRE (tetramethylrhodamine ethyl ester) as per the manufacturer’s instructions. To observe mitochondrial dynamics, cells were incubated with 200 nM MitoTracker Orange CMTMRos, followed by labeling with 2 drops/mL ActinGreen 488 reagent for tracking F-actin and mounted with ProLong Diamond mounting medium containing DAPI for confocal imaging. The fluorescence intensity profiles were plotted using LAS X software (Leica Microsystems). The super-resolution imaging of mitochondria was carried out using a Nikon N-SIM system equipped with an Apochromat 60×/1.27 numerical aperture water-immersion objective len (Nikon Instruments, Tokyo, Japan). Raw SIM images were obtained and reconstructed using Nikon Elements software. The lengths of mitochondria were analyzed by Imaris 8.0 software (Bitplane, Badenerstrasse, Zurich, Switzerland). For the assessment of filopodia assembly, cells were immunostained with anti-VASP antibody followed by Alexa Fluor 488-conjugated secondary antibody. Actin filaments were revealed by Alexa Fluor 594 phalloidin (Invitrogen) staining. The images were taken with the TCS SP5 confocal microscope, followed by fluorescence signal quantification with MetaMorph software (Molecular Devices, San Jose, CA, USA).

### Flow cytometry for cell cycle, mitochondrial ROS, and membrane potential analyses

To characterize cell cycle phase, the collected cells were stained with propidium iodide solution and subjected to a FACSCalibur flow cytometer (BD Biosciences, San Jose, CA, USA). The proportions of cells in different phases of the cell cycle were calculated using CellQuest software (BD Biosciences). The levels of cellular ROS, mitochondrial superoxide, and ∆ψ mt were determined by FACSCalibur and CellQuest using the CellROX Green, MitoSOX Red and TMRE staining, respectively.

### Cell migration and invasion assays

Cell migration was assessed using time-lapse microscopy as previously reported [30] with slight modifications. In brief, approximately 2 × 10^4^ cells in each study group were seeded in 6-well plates in their corresponding pH*e* medium. On the next day, cells were placed on a Leica AF6000 LX microscope and their movement was recorded for 3 h at 20 min intervals at 37 °C. Individual cells were tracked for motility analysis with Image-Pro Plus software (Media Cybernetics, Rockville, MD, USA). To determine cell invasive capability, cells were stained with 1 μM CellTracker Deep Red dye (Invitrogen), and then seeded in 35-mm glass bottom culture dishes (MatTek, Ashland, MA, USA) precoated with Matrigel Matrix (Corning, Tewksbury, MA, USA) supplemented with 25 μg/mL DQ-collagen IV (Invitrogen). The dishes were left for 16-18 h before nuclear staining with 1 μg/mL Hoechst 33342. Images of live-cell invasion were photographed by the TCS SP5 confocal microscope followed by quantitative analysis using Imaris 8.0 software.

### Plasmid construction and RNA interference

To generate HA-tagged constructs, the HA (YPYDVPDYA) epitope-tag was cloned into the pGW1-CMV expression vector (Addgene, Watertown, MA, USA) as a positive control to assess transfection efficiency as previously described [31]. The construction of two sub-clones, pGW1-HA-Drp1^S637A^ and pGW1-HA-Drp1^S637D^, was engineered as detailed elsewhere [32]. Cells were transfected with each of the recombinant plasmids using Lipofectamine 3000 (Invitrogen) according to the manufacturer’s guidelines. For gene-silencing studies, non-targeting control siRNA and sequence-specific SMARTpool siRNA against Mfn2 (see Supplementary Table S2) were purchased from Dharmacon by Horizon Discovery (Cambridge, UK). Transfections were performed using Lipofectamine RNAiMAX transfection reagent (Invitrogen) as per manufacturer’s protocol.

### Western blot

For whole-cell protein extraction, cells were harvested and lysed in 1x RIPA lysis buffer (Merck Millipore) containing PhosSTOP phosphatase inhibitors and cOmplete protease inhibitor cocktail (Sigma-Aldrich by Merck). Protein concentrations were determined by the Pierce BCA assay kit (Thermo Fisher Scientific) using BSA as standard. Equal protein quantities of cell lysates (20-50 μg) were electrophoresed on a range of 10-15% (w/v) SDS-PAGE depending on the size of the target proteins, and then processed for Western blotting with corresponding primary antibodies followed by incubation with HRP-conjugated secondary antibodies. Chemiluminescence signals were visualized using an ECL Western Detection System (Merck Millipore) as recommended by the manufacturer. Unless otherwise specified, β-actin served as loading control in all Western blot experiments.

### Analysis of OPA1 oligomerization

To investigate changes in OPA1 oligomerization correlated with altered cristae ultrastructure, cells were crosslinked with 1 mM bismaleimidohexane for 20 min at 37 °C. After crosslinking, cells were quenched and washed with PBS supplemented with 0.1% β-mercaptoethanol, and were then lysed in RIPA buffer for gradient gel electrophoresis using EVOgel (GeneDireX, Taoyuan, Taiwan). OPA1 oligomerization was analyzed by Western blotting using an anti-OPA1 antibody.

### Microarray and GSEA

Total RNA was extracted using RNeasy mini kit (Qiagen, Hilden, Germany) according to the manufacturer’s guidelines. The quality of RNA was ascertained both by gel electrophoresis and Agilent 2100 Bioanalyzer. Microarray analysis was conducted on the high-resolution Human Transcriptome Array 2.0 platform (Affymetrix, Santa Clara, CA, USA) by the Core Instrument Center at National Health Research Institutes, Taiwan. Raw intensities were normalized using SST-RMA (signal space transformation-robust multi-chip analysis) algorithm. Fold-change values were analyzed by Partek Genomics Suite statistical software (Partek, St. Louis, MO, USA). Gene expression profiles were subjected to GSEA (Gene Set Enrichment Analysis, https://www.gsea-msigdb.org) and IPA (Ingenuity Pathway Analysis, Qiagen) functional enrichment analysis for a variety of gene sets including the pre-defined gene sets, the hallmark gene sets and the representative MSigDB (Molecular Signatures Database) signatures (see Supplementary Table S3). The relative gene expressions of leading edge subsets were viewed as heatmaps by MeV software (https://webmev.tm4.org). The analyzed gene sets were limited to those that contained between 15 and 500 genes. The permutation was conducted 1000 times with random combinations according to default-weighted enrichment statistics, along with the use of a signal-to-noise metric to rank genes based on their differential expression levels across different cell groups.

### Survival analysis

Gene expression data and relevant clinical information were obtained from The Cancer Genome Atlas pancreatic cancer dataset (TCGA_PAAD cohort) downloaded from GDC data portal (https://portal.gdc.cancer.gov). Primary pancreatic tumor samples with available overall survival data (*n* = 176) were selected for analysis. The Kaplan-Meier curve was used to estimate the effects of gene on the overall survival of patients. The patients were classified into low and high expression groups based on the median-centered gene expression values. The difference between low or high expression groups was assessed by log-rank test.

### Statistical analysis

Data are presented as the means ± SD from at least three individual experiments, unless specified otherwise. Results were analyzed by GraphPad Prism statistical software (GraphPad Software, San Diego, CA, USA) using unpaired Student’s *t*-test. A *p* value less than 0.05 was interpreted as statistically significant for all comparisons.

## Results

### Effect of various periods of acidic pH*e* stress on PDAC cell proliferation and viability

**Fig. 1:**
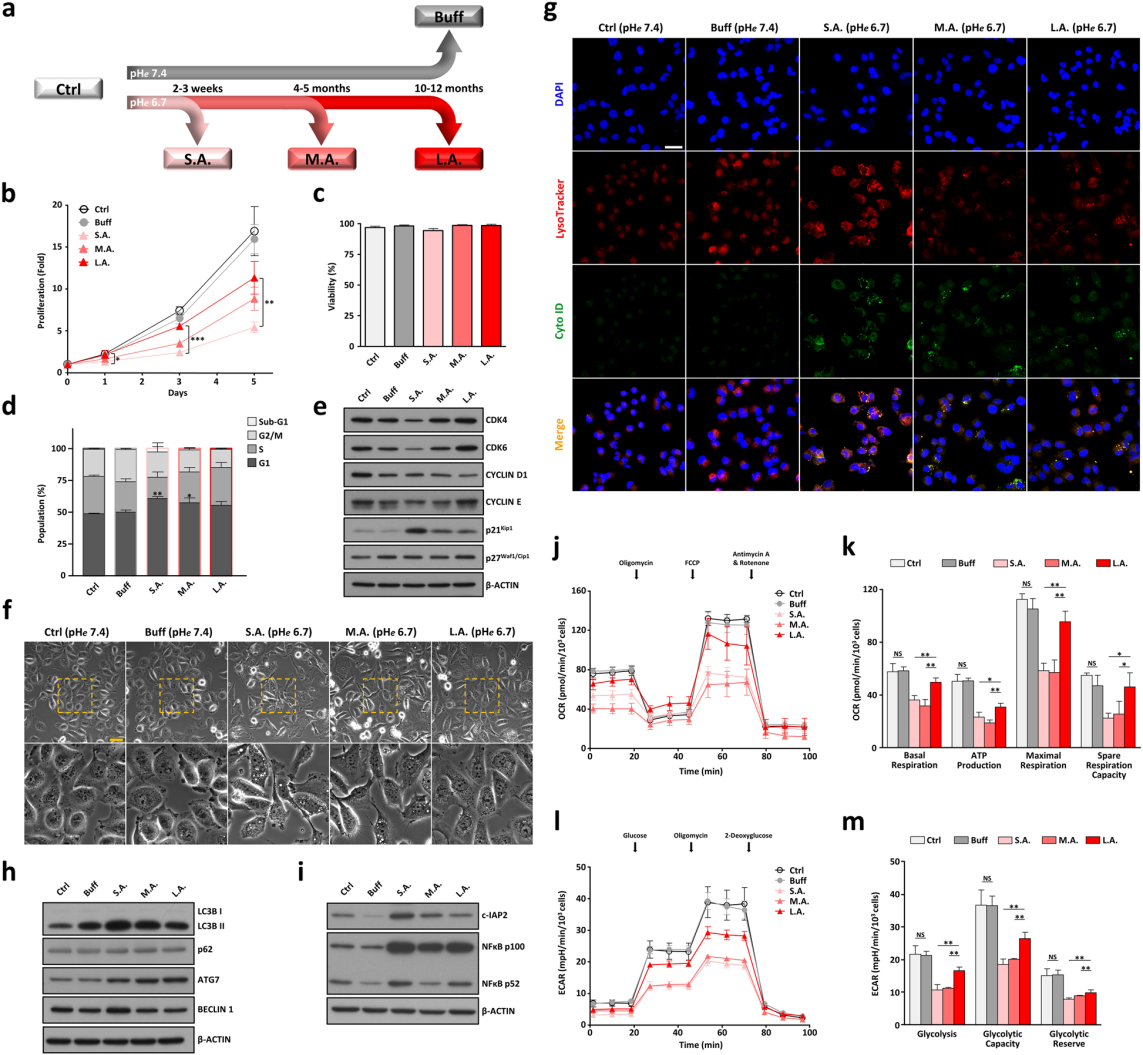
Short-term response and long-term adaptation of PDAC tumor cells to acidic pH*e* stress. **a** Scheme of PDAC cells exposed to various periods of extracellular acidification. Five cell groups were established including the control group (*Ctrl*) cultured in RPMI-1640 medium at pH*e* 7.4; the buffered group (*Buff*) in which cancerous cells were cultivated in RPMI-1640 medium supplemented with HEPES/PIPES buffer at pH*e* 7.4; and the three acid-treated groups with tumor cells incubated in buffered medium at mild pH*e* 6.7 for a short period of 2-3 weeks (*S.A.,* short-term acidification), a medium period of 4-5 months (*M.A.,* medium-term acidification), or a long period of 10-12 months (*L.A.,* long-term acidification). **b** The time effects of acidic pH*e* stress on the proliferation of SUIT-2 cells were evaluated with MTT assay; data are shown as the mean ± SD (*n* = 3) and as fold-change relative to day 0. **c-d** The time effects of extracellular acidification on cell viability and cell cycle progression. Asterisks represent significant differences between the indicated groups versus the *Buff* group (means ± SD, *n =* 3). **e** Western blots of cell cycle-related proteins under various control and acidic pH*e* stress conditions. **f** The time effects of extracellular acidification on cell phenotype and autophagy. The bottom panels display enlargement of the yellow dotted-boxed area from the respective top panels. Scale bar: 50 μm. **g** Accumulation and clearance of autophagic vacuoles and autolysosomes (autophagosome-lysosome fusion) in response to different periods of acidic pH*e* exposure. Scale bar: 50 μm. **h-i** Representative blots of autophagy-related markers and pro-survival proteins. **j-m** Seahorse assays for SUIT-2 cells under various control and acidic pH*e* conditions with oxygen consumption rates (OCR) and extracellular acidification rates (ECAR) displayed as mean ± SD (*n* = 4). FCCP: carbonyl cyanide-4(trifluoromethoxy)phenylhydrazone; NS: not significant (*p* > 0.05); *p < 0.05; **p < 0.01; ***p < 0.001

To investigate the short- and long-term effects of extracellular acidification on pancreatic tumor cells, two representative PDAC cell lines (SUIT-2 & BxPC-3) were subjected to different periods of acidic pH*e* stress. Here, SUIT-2 was utilized as the primary cell line for the majority of studies whereas BxPC-3 served as the second cell line to confirm the key findings and to rule out cell line-specific effects. As illustrated in Fig. 1a, five groups in three replicates per cell line were established including: 1) control group (*Ctrl*) in which PDAC cells were grown in control medium at physiological pH*e* 7.4; 2) buffered group (*Buff*) in which PDAC cells were incubated in control medium supplemented with non-volatile HEPES/PIPES buffer at pH*e* 7.4; and 3-5) three acid-treated groups in which PDAC cells were cultured in control medium buffered with HEPES/PIPES at pH*e* 6.7 for a short period of 2-3 weeks to reflect the initial stage of extracellular acidification (denoted hereafter as *S.A.* for short-term acidic exposure); a medium period of 4-5 months to mirror the reported median TVDT in pancreatic cancer patients [22, 23] (denoted hereafter as *M.A.* for mid-term acidic exposure); or a long period of 10-12 months to simulate the TVDT in patients with early primary lesions [24–26] (denoted hereafter as *L.A.* for long-term acidic exposure). The pH*e* 6.7 represents the modest level of extracellular acidity frequently detected in different types of solid tumors including PDAC [12, 16, 33–37]. Each culture medium was carefully prepared as recommended by Michl et al. [29] with the desired pH*e* value stably maintained within ±0.1 units to ensure quality control and data reliability. Consistent with previous findings [38], we found no substantial effect of salinity on test cells from salt ions present in the mildly acidic medium (data not shown).

Upon initial exposure to acidotic pH*e* stress, the proliferation of SUIT-2 PDAC cells was severely impaired as compared to those grown at pH*e* 7.4 (see *S.A.* vs *Ctrl* or *Buff*, Fig. 1b). The diminished cell proliferation was slightly restored after some generations of acidic exposure (see *M.A.* in Fig. 1b). When cultivated for a longer period up to ~ 1 year, SUIT-2 tumor cells gradually recovered and proliferated at a rate closer, but not yet equal, to those maintained at pH*e* 7.4 (see *L.A.* vs *Ctrl* or *Buff*, Fig. 1b). No apparent reduction in cell viability was detected during the course of the experiment (Fig. 1c). Cell cycle analysis revealed a disproportionate increased percentage of G1 phase and a decreased percentage of S phase in PDAC cells under initial acidic stress (see *S.A.* vs *Ctrl* or *Buff*, Fig. 1d), which indicate a cell-cycle arrest at G1 phase likely induced by acute extracellular acidosis for subsequent cell growth inhibition. This notion was further corroborated by Western blotting: the protein levels of G1-phase regulators (e.g., CDK4, CDK6, Cyclin D1, & Cyclin E) were down-regulated and some CDK inhibitors such as p21^Kip1^ were up-regulated under early acidic stress condition (see *S.A.* in Fig. 1e). After a prolonged acidic exposure, most of the altered expressions in G1/S cell cycle transition regulators were reversed to normal levels, and the high proportion of G1-phase cells was reduced to near, but not equal, to those of the controls at pH*e* 7.4 (see *L.A.* vs *Ctrl* or *Buff*, Fig. 1d-e). Overall, these results point to a slow recovery of the proliferation rate of viable PDAC cells to external acidotic stress stimuli, most likely by progressively overcoming G1 arrest signals to promote cell cycle progression in a time-dependent manner.

### Acute induction of autophagy and EMT as early stress responses followed by their chronic abrogation for long-term cellular adaptation to extracellular acidity

To further assess the differences between short-term response and long-term adaptation of solid tumor cells to extracellular acidification, we examined the phenotypic changes of SUIT-2 tumor cells under various periods of acidic pH*e* challenge. Upon initial acidotic stress exposure, SUIT-2 cells promptly lost their cobblestone appearance with cell-cell contacts and instead induced a spindle phenotype with a concomitant accumulation of autophagic vacuoles (see *S.A.* vs *Ctrl* or *Buff*, Fig. 1f-g). This early phenotypic stress response is similar to previous findings on other types of solid tumor cells [17, 18] that low pH*e* induced an EMT-like process associated with an activation of autophagy to sustain cell viability and growth. Quantification of protein expression revealed an overexpression of LC3B II, Beclin 1, and ATG7 markers with a lack of p62 accumulation (see *S.A.* in Fig. 1h), which confirm the presence of autophagy induction with an enhanced autolysosomal degradation upon acute acidic stress stimuli. Interestingly, SUIT-2 cells reverted to their former phenotype when gradually adapted to the prolonged extracellular acidity (see *L.A.* in Fig. 1f), and the early acidosis-induced autophagic vacuoles and autolysosomes were nearly completely abrogated along with decreased LC3B II and Beclin 1 expression (Fig. 1g-h). This gradual decline in autophagy over time is different from previous reports claiming that the acidosis-induced cell autophagy was constantly active and not reversed [12, 13]. Further western blot analyses showed that, similar to other tumor types studied [39–42], both NF-κB signaling and c-IAP2 expression were significantly higher in the *S.A.* state than in the control states, thus exerting an apoptotic resistance especially under acute acidic stress (Fig. 1i). Since low environmental pH can be viewed as a metabolic stressor and that metabolic changes are often intertwined with the dynamic transition between epithelial and mesenchymal phenotypes [11, 43], our data suggest that tumor cells under acute acidotic pressure rapidly activated EMT and autophagy as early pro-survival responses to overcome metabolic stress and to resist apoptosis. They later developed alternative metabolic strategies to enable their adaptations to a constant and prolonged acidic pH*e* stress.

### Reversible metabolic plasticity as an adaptive strategy in response to extended periods of acidic pH*e* stress

Cellular energy status is an important factor for cell cycle progression, which may be compromised by environmental or metabolic stress that either interfere with ATP production or accelerate ATP consumption [44]. To evaluate the energy metabolic status of SUIT-2 cells under various periods of acidic pH*e* stress, we performed Seahorse extracellular flux assays to determine and compare the oxygen consumption rate (OCR) and the extracellular acidification rate (ECAR) between five different PDAC cell states. Upon early exposure to extracellular acidosis, PDAC cells displayed a substantial decrease in basal respiration, ATP turnover, mitochondrial maximal respiration, and spare respiratory capacity (see *S.A.* vs *Ctrl* or *Buff*, Fig. 1j-k). When SUIT-2 cells were acclimatized to the persistent acidic stress conditions, the initially reduced metabolic parameters caused by acute acidosis were shown to be progressively restored (see *L.A.* in Fig. 1j-k). Real-time ECAR measurements further revealed a similar recovering trend of basal glycolysis and maximal glycolytic capacity after SUIT-2 cells chronically adapted to the prolonged acidic stress stimuli (Fig. 1l-m). Together these results suggest that early acidotic pH*e* stress caused severe mitochondrial damage and ATP shortage. Nevertheless, after being cultivated for a sufficiently long period of time, PDAC cells progressively evolved the ability to reinvigorate mitochondrial activity and to repair dysfunctional metabolism to generate the energy needed for survival and proliferation under an acidic pH*e* microenvironment.

### Atypical mitochondrial network dynamics for early fast response and later slow adaptation to extracellular acidification

**Fig. 2:**
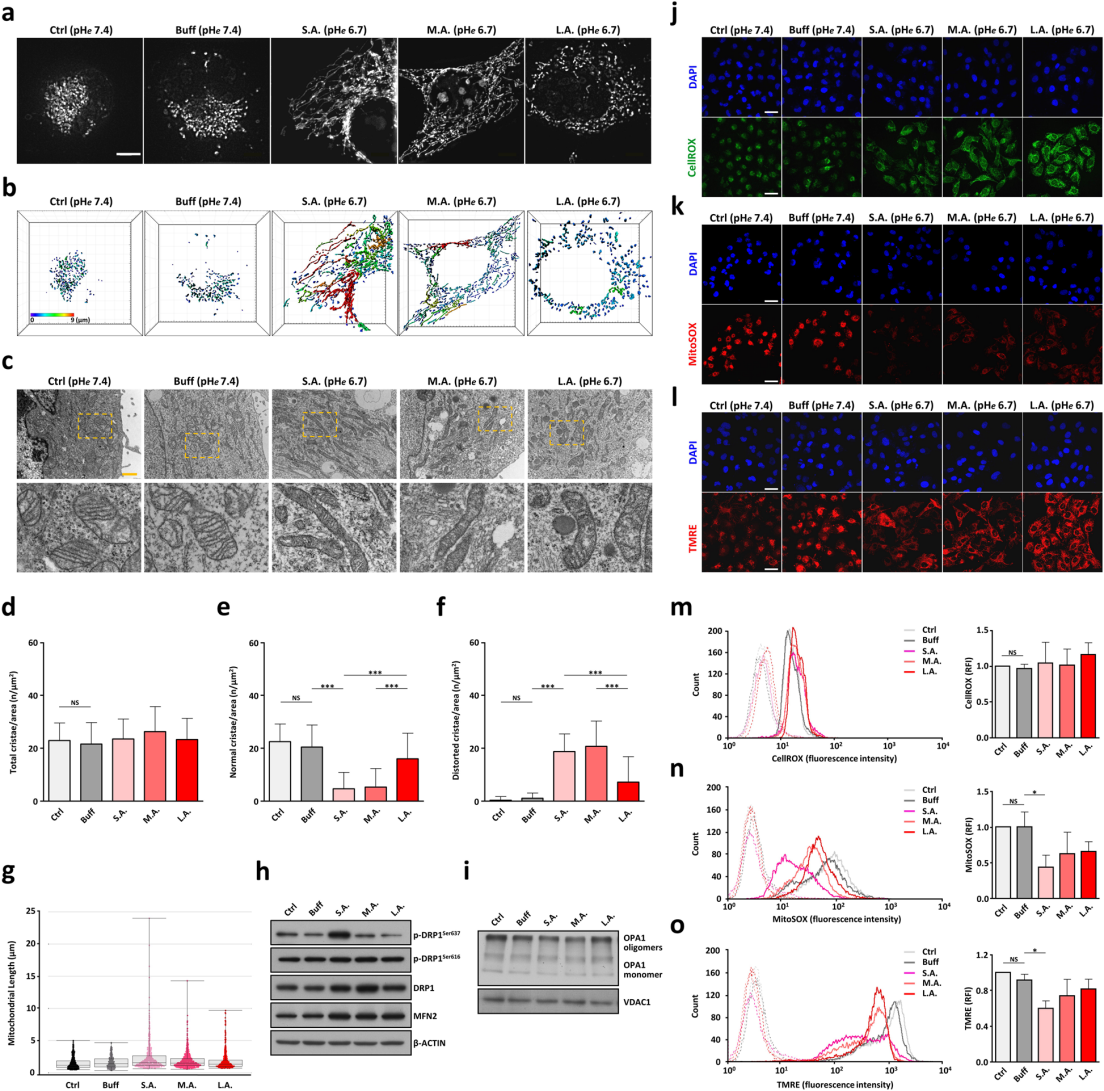
Short- and long-term effects of extracellular acidity on PDAC mitochondrial network dynamics and bioenergetic reprogramming. **a-b** Representative N-SIM images (scale bar, 10 μm) show the time effects of extracellular acidosis on mitochondrial network dynamics in PDAC cells, which were processed by Imaris software for mitochondrial distribution and heterogeneity. The color scale from blue to red represents the different lengths of the mitochondrial tubules between branch points (0 to 9 μm). **c** TEM images of the time effects of acidic pH*e* stress on mitochondrial cristae ultrastructure. The lower panels are magnified areas derived from the respective upper panels as indicated by the boxed region. Scale bar: 2 μm. **d-f** The dynamic nature of cristae remodeling analyzed by ImageJ software with three column charts representing the number of total cristae, normal cristae, and distorted cristae per unit of mitochondrial cross-sectional areas as mean ± SD (*n* = 45 mitochondria). **g** Quantification of mitochondrial morphology from N-SIM images with three cells per condition randomly selected and combined to draw the full range of mitochondrial length distributions. **h** Representative blots of mitochondrial dynamics-related proteins under different control and acidic pH*e* stress conditions. **i** Western blots of OPA1 oligomerizations with VDAC as the loading control. **j-o** Cellular ROS, mitochondrial superoxide, and membrane potential were analyzed by confocal microscopy (**j**-**l**) and flow cytometry (**m**-**o**) using CellROX Green, MitoSOX Red, and TMRE Red probes, respectively. The relative fluorescence intensity of tumor cells in the absence (dotted line) or presence (solid line) of probes was shown in the histograms on the left. The corresponding values of fluorescence intensity were presented in the right column graphs as mean ± SD (*n =* 3). Scale bar, 50 μm. NS: *p* > 0.05 (not significant), *p < 0.05, ***p < 0.001

Mitochondria are highly dynamic organelles that can sense stress signals and rapidly remodel their structure and network [45–47]. Here, we present evidence of an unusual mode of mitochondrial network dynamics highlighted by an early rapid response and a later slow adaption to constant acidotic pH*e* stress. Super resolution 3D N-SIM microscopy showed the mitochondria of control SUIT-2 tumor cells at pH*e* 7.4 as speckled and spherical nucleus-surrounding clusters (see *Ctrl* and *Buff* in Fig. 2a-b & Videos S1-S2). When exposed to acute extracellular acidosis, however, the mitochondria of SUIT-2 cells rapidly fused into distinct filamentous reticular networks termed as SIMH (stress-induced mitochondrial hyperfusion, [48]) and spread extensively throughout the cytoplasm (see *S.A.* in Fig. 2a-b & Video S3).

After a prolonged period of acidic pH*e* exposure, the hyperfused mitochondrial network underwent a slow progressive ultrastructural transformation from the interconnected reticular SIMH to the punctate fragmented disconnected morphology scattered over the cytoplasm (see *M.A.* and *L.A.* in Fig. 2a-b & Videos S4-S5). This characteristic reorganization of mitochondrial architecture was further confirmed by TEM. The prominent elongated mitochondria with abnormal cristae were promptly induced by acute acidotic stress (see *S.A.* in Fig. 2c), but were slowly restructured into a nearly normal configuration after SUIT-2 cells steadily habituated to the acidic pH*e* microenvironment (see *L.A.* in Fig. 2c). The acid-induced cristae modulation was illustrated by ImageJ software analysis showing a significant decrease in normal cristae and a large increase in distorted cristae per surface area of mitochondria (Fig. 2d-f). Under extended acidic conditions, the distorted cristae ultrastructures were progressively reversed to a near normal status as those seen at pH*e* 7.4 (Fig. 2d-f). The full-range mitochondrial length distributions of five SUIT-2 cell states were analyzed by Imaris software. The data showed a mitochondrial network hyperfusion with a wide range of mitochondrial lengths as an early responsive machinery (see *S.A.* in Fig. 2g). This was followed by a mitochondrial network fragmentation with shorter mitochondrial lengths as a late adaptative mechanism to the prolonged acidic pH*e* pressure (see *L.A.* in Fig. 2g).

The dynamism and health of the mitochondrial network is delicately regulated by fusion/fission events [45–47]. Figure 2h shows that there is a significantly elevated expression of inactive form p-DRP1^Ser637^ detected exclusively in the *S.A.* cell state in addition to an increase of DRP1 total protein in three acid-treated cell states. The increased phosphorylation at serine 637 of fission protein DRP1 suppressed its translocation to the mitochondria [46, 47], which enhanced mitochondrial fusion and prevented mitochondria from autophagic degradation (Fig. 2a-b & Video S3). Notably, after an extended exposure to the same acidotic pH*e* stress, the expression of p-DRP1^Ser637^ was reversed to a level similar as control cells at pH*e* 7.4 (Fig. 2h), thus leading to an activation of mitochondrial fission to trigger mitochondrial fragmentation (Fig. 2a-b & Video S5). A simultaneous up-regulation of mitochondrial pro-fusion protein MFN2 was also seen in the *S.A.* cell state, which enables more fusion activity by tethering adjacent mitochondria together under early acidic pH*e* pressure (Fig. 2h). Furthermore, the tightness of mitochondrial cristae junctions has been reported to be regulated by the oligomerization of long and short forms of cristae-remodeling protein OPA1 [46, 47]. Hence, the decrease of OPA1 oligomerization detected in the *S.A.* cell state points to a marked loss of normal cristae structures; in the *L.A.* cell group many distorted cristae were steadily restored to normal and functional configurations (Fig. 2c-f & i).

Oxidative stress refers to elevated intracellular levels of reactive oxygen species (ROS). It is a common issue for cancer cells under external stimuli because of its ability to initiate cell death [46]. Considering that we did not observe any substantial reduction in cell viability during the mild acidic stress exposure (Fig. 1c), we attempted to assess and compare the status of oxidative stress among five different SUIT-2 cell states. Herein, oxidative stress was measured based on the levels of cellular ROS, mitochondrial ROS, and mitochondrial membrane potential using CellROX Green, MitoSOX Red, and TMRE fluorescent probes, respectively. Figure 2j & m shows that no apparent difference was found for the production of overall cellular ROS in SUIT-2 cells under normal or acidic pH*e* conditions. Compared to control cells at pH*e* 7.4, however, there was a statistically significant inhibition of both the MitoSOX-measured superoxide and the TMRE-measured membrane potential in PDAC cells under acute acidotic stress (see *S.A.* in Fig. 2k, l, n, & o). These data strongly suggest that PDAC cells rapidly reduced oxidative phosphorylation and lowered mitochondrial ROS production in response to acute extracellular acidosis.

### Perturbation in mitochondrial network dynamics leads to poor response to acute acidic pH*e* stress

**Fig. 3:**
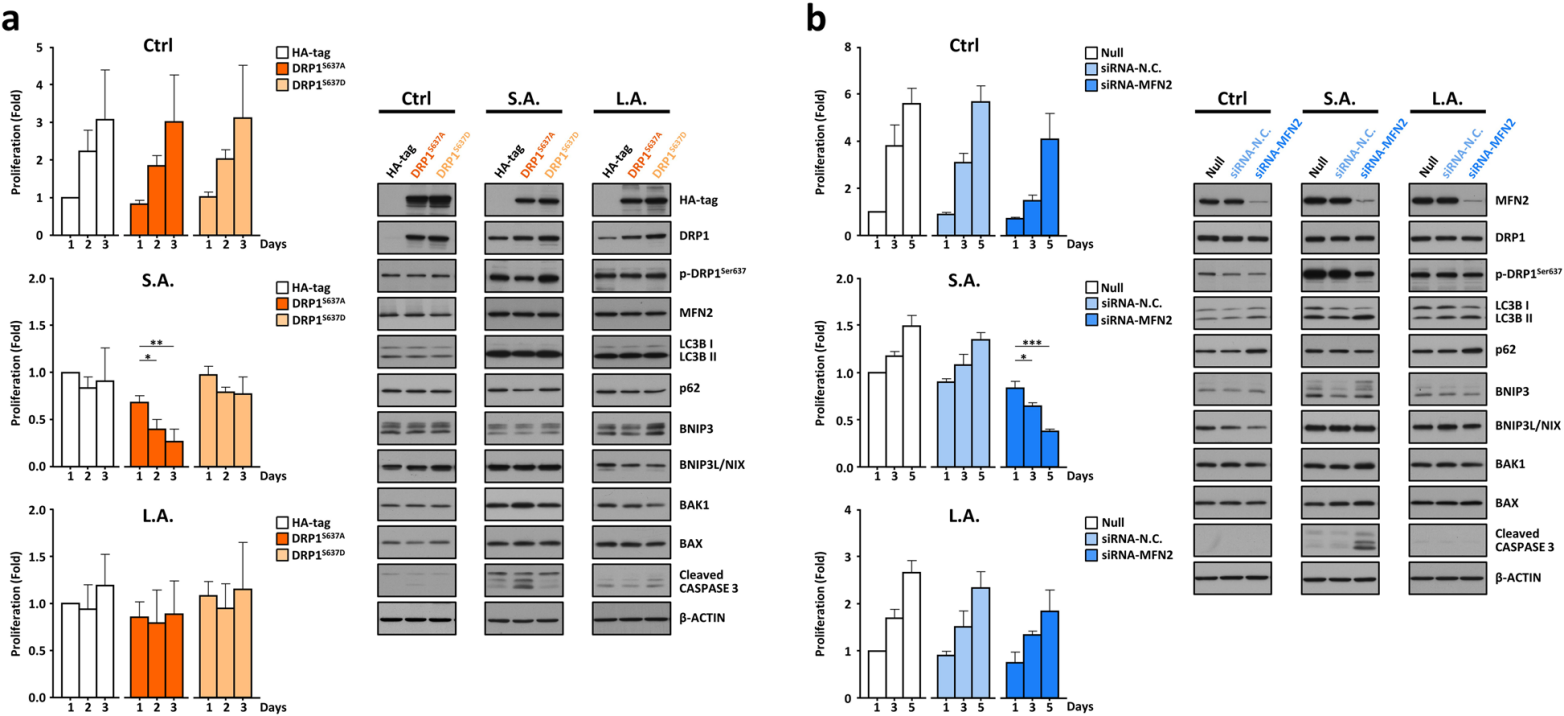
Disturbance in PDAC mitochondrial network dynamics leads to poor response to initial extracellular acidotic stress. **a** SUIT-2 PDAC cells under different control and acidic stress conditions (*Ctrl*, *S.A.,* & *L.A.*) were individually transfected with the control HA-tagged plasmid, the phosphodeficient DRP1^S637A^ mutant, or the phosphomimetic DRP1^S637D^ variant. MTT proliferation assays (mean ± SD, *n =* 3, fold-change relative to day 1) and Western blot analyses were performed to assess the impact of altered DRP1 fission activity in disrupting mitochondrial network dynamics involved in the early response and late adaptation to acidic pH*e* stress. **b** The same three SUIT-2 PDAC cell groups (*Ctrl*, *S.A.,* & *L.A.*) were individually transfected with either negative control non-targeting siRNAs (siRNA-N.C.) or siRNAs against MFN2 (siRNA-MFN2). Tumor cells from each group without siRNA transfection served as null controls. The mean proliferation of each cell group was determined by MTT assays (mean ± SD, *n =* 3, fold-change relative to day 1), and the expression changes in mitophagy- and apoptosis-related molecules were determined by Western blot analyses; β-actin was the loading control

Increased ROS produced by damaged mitochondria can trigger mitophagy—a crucial mechanism for regulating mitochondrial quality and cellular homeostasis especially under stress [45]. SUIT-2 PDAC cells could induce massive SIMH to prevent mitochondria from mitophagic degradation by steric obstruction (Fig. 2a-h & Video S3). Thus, we manipulated DRP1 fission and MFN2 fusion activities to disintegrate mitochondrial hyperfusion and promote selective mitophagy that ultimately leads to the disruption of tumor cell adaptation to extracellular acidosis. As presented in Fig. 3a, we individually overexpressed the control HA-tagged protein, the phosphodeficient DRP1^S637A^, or the phosphomimetic DRP1^S637D^ variant [31] in three different cell states (*Ctrl*, *S.A.,* & *L.A.*). Despite the modest cytotoxicity to the infected cells, we found that overexpression of the DRP1^S637D^ variant had no statistical difference in terms of MTT proliferation assay versus those overexpressing the HA-tagged protein regardless of culture in physiological pH*e* 7.4 or mild acidic pH*e* 6.7 (Fig. 3a). In contrast, overexpression of the DRP1^S637A^ mutant in the *S.A.* cell group resulted in more severe cell death than the *Ctrl* and *L.A.* cell groups (Fig. 3a). A lower amount of phosphorylated DRP1^S637^ was also detected in the S.A. cell group overexpressing phosphodeficient DRP1^S637A^. This synergistically enhanced DRP1 fission activity fragmented the hyperfused mitochondria followed by selective mitophagy (e.g., differential expressions of LC3B II, BNIP3, & BNIP3L/NIX) and apoptotic induction (e.g., elevated levels of BAK1, BAX, & Cleaved Caspase 3) (see *S.A.* vs *Ctrl* in Fig. 3a). Notably, overexpression of the same DRP1^S637A^ variant in the *L.A.* cell state did not induce mitophagy or apoptosis probably due to the presence of restored fragmented and relatively healthy mitochondria in long-term acid-adapted PDAC tumor cells.

To obtain more convincing evidence on the impact of perturbing dynamic mitochondrial networks on tumor cell responsiveness to acidic pH*e* stress, we used a more efficient and less cytotoxic approach to transfect the same three SUIT-2 cell states (*Ctrl*, *S.A.,* & *L.A.*) with either negative control non-targeting siRNAs (siRNA-N.C.) or siRNAs against MFN2 (siRNA-MFN2). Unlike the control siRNA-treated cells, knockdown of MFN2 expression with a parallel decrease of phosphorylated DRP1^Ser637^ in the *S.A.* cell group led to a reduced mitochondrial fusion and an enhanced mitochondrial fission that collectively enabled selective mitophagy (e.g., up-regulation of LC3B II & BNIP3) followed by cell apoptosis (e.g., increased levels of BAK1, BAX, & Cleaved Caspase 3) (see *S.A.* in Fig. 3b). In contrast, there was no significant inhibitory effect of siRNA-MFN2 on cell proliferation nor was there impaired viability in the *L.A.* cell group (see *L.A.* in Fig. 3b). These data highlight the critical role of mitochondrial network dynamics for the systematic response and adaptation of PDAC cells to the external acidic stimuli. A disruption in this dynamic process severely hampered the capability of pancreatic tumor cells to deal with the acidified microenvironment. This subsequently caused an inadequate response or intolerance to the stress of extracellular acidosis especially under acute exposure situation. However, PDAC cells develop a near normal active metabolic state via a slow mode of mitochondrial network reorganization after a constant and prolonged acidic exposure. At this long-term acid-adapted *L.A.* cell state, the same strategy of manipulating fission/fusion proteins to fragment the already fragmented mitochondria may not be as effective as it was to the hyperfused mitochondria present in the early acidity-responsive *S.A.* cell state.

### Short- and long-term effects of extracellular acidification on mitochondrial motility and metastatic potential

**Fig. 4:**
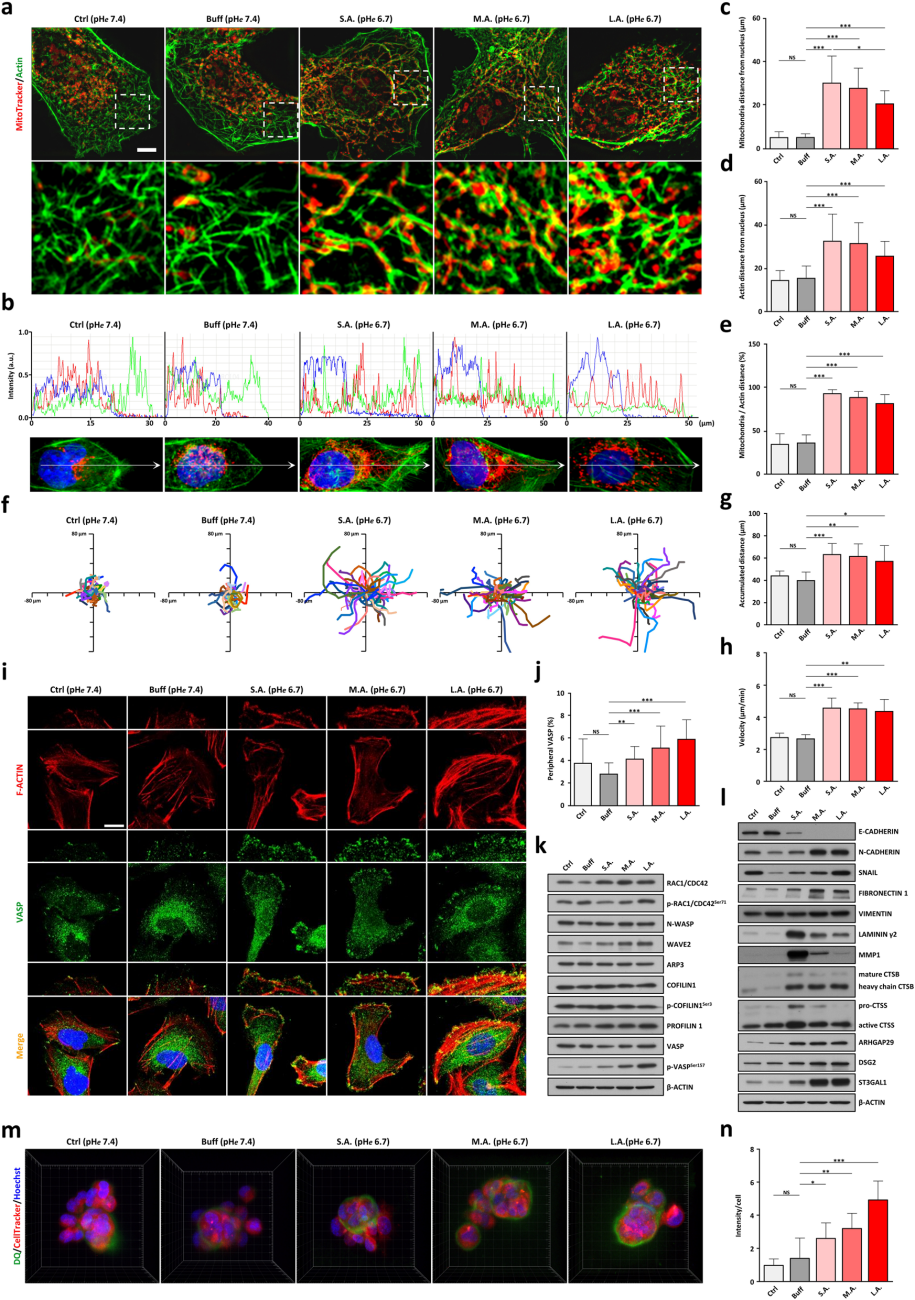
Short- and long-term effects of extracellular acidification on PDAC mitochondrial movement, cell migration, and invasion. **a** Top: N-SIM images (scale bar, 5 μm) show the time effects of extracellular acidity on mitochondrial movement and distribution in SUIT-2 PDAC cells. Bottom: magnified images of colocalization of mitochondria (MitoTracker Red) and F-actin (ActinGreen 488) from the dotted boxed area in the respective top panels. **b** Graphs represent the normalized fluorescence intensity calculated on images obtained from cells immunostained for mitochondria (MitoTracker Red), F-actin (ActinGreen 488), and nucleus (DAPI Blue) along the line arrows from the nucleus to the cell edge in micrometers. **c-e** Column graphs quantify the distance from the nucleus to mitochondria, the distance from the nucleus to the actin filaments, and the relative motility ratio. Data are represented as mean ± SD (*n* > 10). **f** Representative tracings of 10 randomly migrating cells per group. Distance scale is in micrometers. **g-h** The time effects of extracellular acidosis on the accumulated distance and velocity of tumor cell movement were summarized as mean ± SD (*n* ≥ 40). **i** VASP and F-actin were respectively immunolocalized with anti-VASP antibody (green) and Alexa Fluor 594 phalloidin (red). Insets are enlarged images of peripheral areas of cells. Scale, 10 μm. **j** The ratio of VASP in the peripheral region was expressed as a mean ± SD (cell number, *n* ≥ 10). **k** Western blots of actin filament assembly and turnover regulators. **l** Western blots of invasion-related markers. **m** Degradation of DQ-collagen IV (green) in live PDAC cells (red) under extracellular acidity. **n** Column charts represent the fluorescence intensity of degraded DQ-collagen IV per cell (mean ± SD). At least seven clusters per cell group were included for quantification. *p < 0.05, **p < 0.01, ***p < 0.001

Accumulating evidence links mitochondrial subcellular distribution to tumor cell morphology, proliferation, and metastatic potential [49]. Figure 4a shows that many mitochondria in SUIT-2 PDAC cells under acidic pH*e* stress moved from near the nucleus to the cell periphery via anchoring to actin filaments. The elevated fluorescence intensity of MitoTracker (red) along the arrows (white) indicated an anterograde movement of mitochondria toward the plasma membrane (Fig. 4b). Statistical analysis of the quantification of the mitochondrial moving distances also validated such phenomenon (Fig. 4c-e). Interestingly, when SUIT-2 cells acclimated to the constant stress of extracellular acidity, many mitochondria were fragmented from reticular SIMH (Fig. 2a-b) but still remained at the leading edge of the cell periphery (Fig, 4a-e). This scene was most likely to support ATP-consuming activities for cell membrane dynamics, migration, and invasion.

Tumor cell migration and invasion were recently proposed to be regulated by mitochondrial trafficking [49]. By performing time-lapse imaging to track the migration of individual SUIT-2 cells exposed to different periods of extracellular acidity, we found that solid tumor cells under acute acidic pH*e* are more prone to adopt to faster migration than those at control pH*e* 7.4 (see *S.A.* vs *Ctrl* or *Buff*, Fig. 4f). This migration ability remained robust when PDAC cells further adapted to the prolonged acidic pH*e* stress (see *M.A. & L.A.* in Fig. 4f). Statistical analysis of the migration distance and velocity confirmed that SUIT-2 cells under pH*e* 6.7 had a higher motility than those under pH*e* 7.4 (Fig. 4g-h). Confocal images of endogenous VASP—a vasodilator-stimulated phosphoprotein essential for filopodia formation—revealed that it was more localized in a line along the tips of the protruding membrane. This presentation suggested an augmented filopodia formation induced by acidotic stress (see Fig. 4i-j). This increased filopodia formation was supported by high levels of actin nucleation and polymerization markers including WAVE2, Profilin 1, phosphorylated VASP^Ser157^, and RAC1/CDC42^Ser71^ (see *L.A.* in Fig. 4k). Remarkably, E-cadherin was almost completely abolished during acidic exposure whereas a drastically enhanced N-cadherin expression was detected when SUIT-2 cells progressively adapted to the constant acidic pH*e* conditions (Fig. 4l). Other transcription factors and mesenchymal markers like SNAIL, Fibronectin 1, and Vimentin also had a differential expression pattern similar to that of N-cadherin, indicating a persistent EMT with epithelial cobblestone phenotype (Fig. 1f) likely associated with cellular adaptation to the prolonged acidic pH*e* exposure (Fig. 4l). Extracellular matrix glycoproteins such as Laminin-γ2 and proteolytic enzymes such as MMP1, CTSS & CTSB were radically up-regulated under acute acidotic stress, but all become less expressed when tumor cells steadily adapted to extended extracellular acidity (Fig. 4l). Some metastasis-related factors including ARHGAP29, DSG2, and ST3GAL1 were up-regulated after an extended exposure to acidic pH*e* (Fig. 4l). Further quantification of protein expression of autophagy, pro-survival, mitochondrial dynamics, and metastasis-related markers showed that many of these critical regulators were differentially expressed within the first few days of acute exposures to extracellular acidosis (Fig. S1). Together these findings demonstrate that PDAC cells rapidly increased mitochondrial motility under acidic stress. They had a more aggressive potential even though their proliferation rate was strongly reduced and most mitochondria were in a hyperfused state associated with ATP insufficiency. By further incubating SUIT-2 cells in 3D overlay culture, we observed an extensive pericellular degradation of DQ-collagen IV under acidic pH*e* conditions especially at the *L.A.* cell state (Fig. 4m-n). These data indicate that PDAC tumor cells became more malignant with increased migratory and invasive potentials when adapted to continuous long-term extracellular acidity.

### Validation of short- and long-term impacts of acidic pH*e* stress on second PDAC tumor cell line

BxPC-3 cells were used as the second PDAC cell line to validate the aforementioned observations of the short-term response and long-term adaptation of SUIT-2 cells to the acidic pH*e* microenvironment. As shown in the *S.A.* state of Fig. S2, upon initial exposure to acidotic stress, BxPC-3 cells also exhibited impaired proliferation (Fig. S2a), increased G1 phase arrest (Fig. S2b), induced EMT phenotype (Fig. S2c), activated cytoprotective autophagy (Fig. S2d), diminished metabolic capacity (Fig. S2e-S2h), stimulated mitochondrial hyperfusion (Fig. S2i-S2j) and enhanced invasive property (Fig. S2k) that are similar to those observed in the S.A. state of SUIT-2 cells. Consistent with the primary SUIT-2 cell line, after BxPC-3 cells progressively habituated to the prolonged acidic pH*e* exposure, many of the altered phenomena were reversed to close to those of the controls at pH*e* 7.4 except with more aggressive potentials with increased invasiveness (see *L.A.* in Fig. S2a-S2k). In should however be noted that, while the majority of differential protein expression patterns were similar between the two cell lines, the autophagy-related markers in BxPC-3 cells appear to be less susceptible to induction by acute extracellular acidity (see *S.A.* vs *Ctrl* or *Buff*, Fig. S2l). These observations are possibly due to the higher basal levels of endogenous autophagy in parental cells (see *Ctrl* in Fig. S2l), so that when exposed to acidic pH*e* the degree of induced activation of protective autophagy to mitigate acidotic stress to sustain cell survival is less drastic in BxPC-3 cells than that in SUIT-2 cells.

### Identification of key factors associated with long-term cellular adaptation to extracellular acidotic stress

The maintenance of stable medium pH is critical for data reproducibility and reliability. However, as recently stated by Michl and co-workers [29], there are still no consensus guidelines for best practices in controlling pH in live-cell culture systems. In this study, we prepared and constantly monitored incubation media pH as per recommendations by Michl et al. [29] and carried out principal component analysis (PCA) for both the untreated and acid-treated PDAC cell groups to determine the key factors involved in adaptation to extracellular acidosis as well as the potential buffer effects from acidic pH*e* cultures. Figure S3a shows that the PCA plot displayed a high degree of concordance between the sample duplicates with two relatively distinctive subpopulations of data points representing a substantial difference in gene expression between PDAC cells at physiological pH*e* 7.4 and mild acidic pH*e* 6.7. Further statistical analysis on the *Ctrl* and *Buff* cell groups revealed that out of 67,528 gene probes analyzed, 99.48 and 99.06% of probes remained unchanged in expression in PDAC cells under short- or long-term buffering conditions, respectively (Fig. S3b). Even after nearly 1 full year of continuous cell culture passage, only 0.39% of probes were up-regulated (fold change ≥2); 0.55% were down-regulated (fold change ≤0.5) to statistical significance. These results—together with the very slight differences in cell phenotype, proliferation, cell cycle phase, and energy metabolic status in between the control and buffer-treated PDAC cell populations (see *Ctrl* vs *Buff*, Figs. 1b, d, j-m, 2a-g, & j-o)—indicate that the diverse short-term responses and long-term adaptations of tumor cells to external stimuli do not result from the effect of the buffering regime but are rather due to the acidic pH*e* stress. Hence, our work strongly supports the guidelines of Michl et al. [29] for proper management of medium pH, which would be advantageous for researchers in the field to minimize untoward pH fluctuations and erroneous inferences for assessing the impact of extracellular acidification on test cells regardless of the short- or long-term culture.

**Fig. 5:**
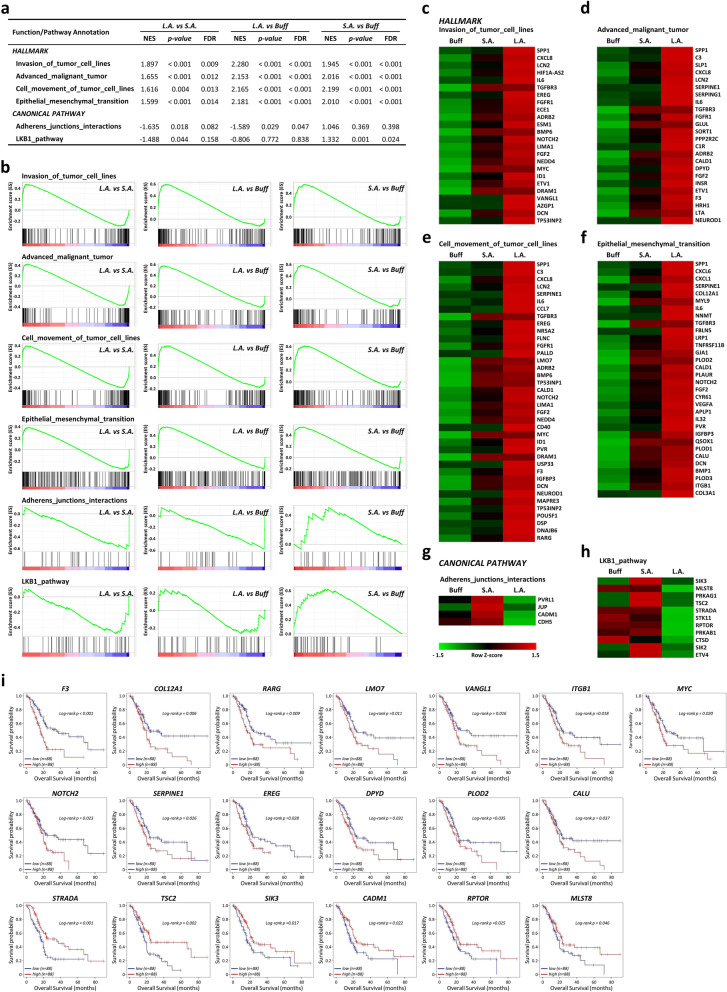
GSEA analysis showing significant enrichment of genes progressively adapted to the sustained acidic pH*e* stress. **a** Table showing six significant functional classes and pathways identified in SUIT-2 PDAC cells progressively adapted to acidotic pH*e* stress. The normalized enrichment scores (NES), *p*-values, and false discovery rates (FDR) obtained from the comparison results of the *L.A.* vs *S.A.* cell group; the *L.A.* vs *Buff* cell group; and the *S.A.* vs *Buff* cell group were listed. **b** Enrichment score (ES, green color) plots of GSEA-extracted representative pathways from microarray data. Six functional pathways were markedly enriched upon tumor cell adaptation to sustained acidic pH*e* stress (see comparisons of the *L.A.* vs *S.A.* group; the *L.A.* vs *Buff* group; and the *S.A.* vs *Buff* group). The gene sets of six functional categories were ranked according to the NES and FDR values. Negative and positive ES values point to gene sets over-represented in the topmost down-regulated or up-regulated genes in acid-adapted cells. Vertical black bars in lower areas refer to individual genes in a functional category, and their position reflects the contribution of each gene from the Human Transcriptome Array 2.0 to the ES. Genes that belong to the leading-edge subset (i.e., genes that appear at or after the ES minimum and at or before the ES maximum) contribute to the enrichment signal. **c-h** Heatmaps represent differential expression of genes belonging to the leading-edge subset of the indicated functional categories. **i** Kaplan-Meier survival analysis of 19 genes identified in the leading edge of the six aforementioned functional categories. The mRNA expression levels were significantly related to PDAC patient survivals in TCGA-PAAD cohort data. All *p*-values correspond to the log-rank test comparing the survival curves

We next compared the global gene expression profiles with public expression databases and determined several critical gene signatures linked to PDAC cell adaptation to the acidic pH*e* environment. There were four functional hallmarks (invasion_of_tumor_cell_lines; advanced_malignant_tumor; cell_movement_of_tumor_cell_lines; and epithelial_mesenchymal_transition) and two canonical pathways (adherens_junctions_interactions and LKB1_pathway) identified from the comparisons between each of *Buff*, *S.A.,* and *L.A.* cell populations (Fig. 5a-b). This analysis suggested that the activation states of the four functional classes are selectively enhanced along with extended periods of tumor cell exposure to acidic conditions whereas the LKB1_pathway is primarily involved in metabolism and growth control [50] during early extracellular acidosis. The adherens_junctions_interactions pathway is predominantly suppressed when PDAC cells adapt to the long-term acidic pressure. These hallmark signatures agree with those shown in Figs. 1 & 2 in that the proliferation and energetic metabolism of PDAC cells were largely restricted when first exposed to extracellular acidosis, but became progressively more aggressive and malignant after long-term adaptation to the acidic pH*e* stress (see Fig. 4). Further heatmap analysis revealed that the top differentially expressed genes belonged to the leading-edge subsets of the indicated functional categories (Fig. 5c-h), suggesting these genes may be involved in the early response or late adaptation of PDAC cells to extracellular acidosis. Kaplan-Meier survival curves on these leading-edge subsets disclosed a total of thirteen acid-adaptation up-regulated genes (*F3, COL12A, RARG, LMO7, VANGL1, ITGB1, MYC, NOTCH2, SERPINE1, EREG, DPYD, PLOD2,* & *CALU*) and six down-regulated genes (*STRADA*, *TSC2*, *SIK3*, *CADM1*, *RPTOR,* & *MLST8*) whose expression levels are respectively negatively and positively correlated with the overall survival of pancreatic cancer patients in the TCGA-PAAD cohort (Fig. 5i).

**Fig. 6:**
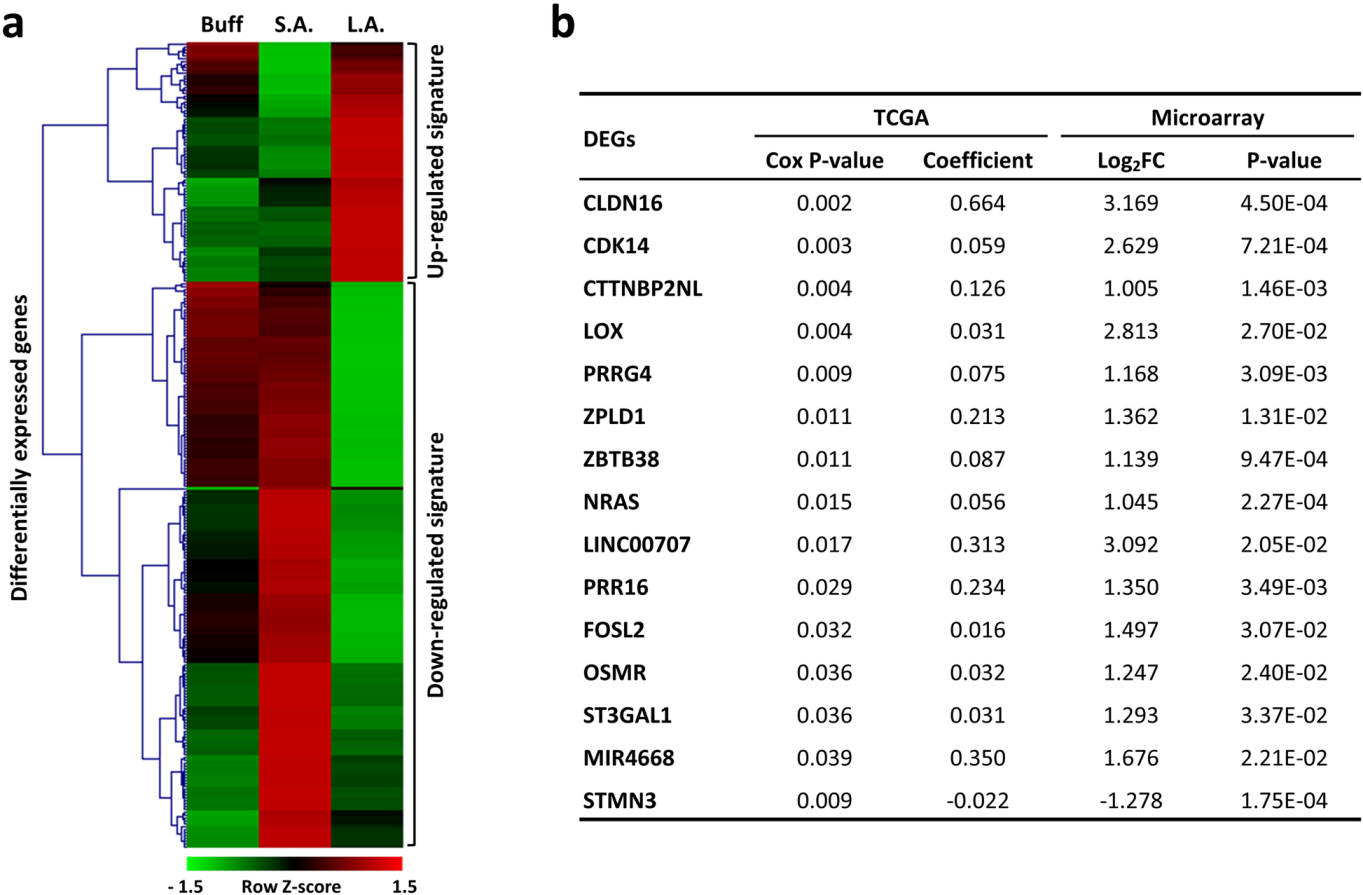
Genome-wide identification and differential expression of signature genes related to chronic adaptation to acidic microenvironment. **a** Hierarchical clustering heatmap of differentially expressed genes in SUIT-2 PDAC cells subjected to acute (*S.A.*) or chronic (*L.A.*) continuous acidotic pH*e* stress. There were 314 genes with significantly differential expression patterns (*L.A.* vs *S.A.*, absolute log_2_ fold-change ≥1, *p* < 0.05) identified and annotated either as a chronic_acidosis_adaptation up-regulated signature or chronic_acidosis_adaptation down-regulated signature. The distance used for the clustering is based on the classical Euclidean distance that allows clustering gene expression by expression levels. Note that very few genes have a differential expression pattern and are significant in the buffer (*Buff*) group; these were excluded. **b** A total of 15 differentially expressing genes (DEGs) identified from the list of chronic_acidosis_adaptation signatures that are significantly related to the overall survival of 176 pancreatic cancer patients in TCGA-PAAD cohort in a Cox regression model

To highlight the molecular targets that are specifically involved in the long-term adaptation of PDAC tumor cells to the acidic pH*e* microenvironment, we analyzed and compared mRNA differential expression profiles between *L.A.* and *S.A.* cell states. Two distinct gene sets were created and annotated for functional inference: the chronic_acidosis_adaptation up-regulated signature (see Table S4) and the chronic_acidosis_adaptation down-regulated signature (see Table S5). Their expression levels are differentially impacted by long-term cell adaptation to extracellular acidosis. The differential expression patterns of the defined gene sets were visualized as a heatmap in Fig. 6a with a total of fourteen up-regulated genes (*CLDN16*, *CDK14*, *CTTNBP2NL, LOX, PRRG4*, *ZPLD1*, *ZBTB38*, *NRAS*, *LINC00707*, *PRR16*, *FOSL2*, *OSMR*, *ST3GAL1*, & *MIR4668*). One down-regulated gene (*STMN3*) was further identified and significantly associated with the overall survival of pancreatic cancer patients (Fig. 6b). These fifteen genes, together with those nineteen genes identified from GSEA analysis in Fig. 5, may represent the central molecular targets to help clarify how solid tumor cells such as PDAC respond and adapt to the enduring acidotic pH*e* stress and then proceed to more aggressive and metastatic stages.

## Discussion

Most solid cancers are asymptomatic in their early stages, with a slow TVDT of months to years depending on primary tumor size and location [25, 26, 51]. Given the long latency of tumor development, the acidification of the extracellular environment is thought to be a lengthy and continuing process. However, most studies to date have only examined the short-term response but not the long-term impact of microenvironmental acidification on solid cancers including PDAC. Even more confusing is that the definition of short term versus long term is not always clear, and can at times be very subjective (e.g., few reports describe an 8-h acidic cell culture as short-term and a 24-h time frame as long-term exposure [13, 14]). Since the tumor growth rate is a key parameter in assessing the malignant potential and therapeutic response of the disease, here we recommend the use of clinical TVDT as a practical reference for investigating the time effects of acidic pH*e* stress on solid tumors. In the current setting, we exposed PDAC cells to different periods of extracellular acidity as illustrated in Fig. 1a. By characterizing these different PDAC cell states under short-, mid-, and long-term acidic stress conditions, we could identify a previously unreported slow-mode of reversible adaptive plasticity of pancreatic tumor cells to the prolonged acidic pH*e* microenvironment. More studies on the long-term impact of extracellular acidosis on other types of solid tumors are currently underway, and will be reported in due course.

Some previous short-term studies claimed that the inhibited cell proliferation induced by acute extracellular acidosis could soon be completely restored under the same acidic culture condition [38]. However, we did not observe any such phenomenon. Rather, we identified a slow but partial recovery of PDAC cell proliferation even after nearly a full year of acidic pH*e* exposure (Figs. 1b & S2a). We showed that the acute extracellular acidotic stress induced a G1 cell cycle arrest that led inhibited cell proliferation. After PDAC cells chronically adapted to the prolonged acidic pH*e* pressure, most of the altered expression in G1/S regulators was reversed to normal levels, and the increased percentage of G1-phase cells was gradually reduced to near, but not equal, to those of controls at pH*e* 7.4 (Figs. 1d-e, S2b, & S2l). The slow mode of reversible recovery in cell cycle progression and proliferation was further underlined by findings showing that PDAC cells rapidly reduced the levels of oxidative phosphorylation and mitochondrial ROS to sustain their survival and minimum growth upon acute acidosis exposure. This continued until they evolved the ability to reinvigorate mitochondrial activity, repair dysfunctional metabolism, and thus generate the energy needed for full proliferation under acidic conditions (Figs. 1j-m, 2j-o, & S2). Such a multistep process would inevitably require substantial time for cells to evolve and adapt to the hostile acidic microenvironment, thus presenting an interesting paradox of how tumor cells could restore full proliferation in just a few days as described previously under early acute acidotic stress [38].

Tumor cells have evolved autophagy and EMT as two major mechanisms to respond to external stress stimuli [52]. Autophagy enables tumor cells to survive environmental stresses by recycling intracellular components to sustain metabolic homoeostasis. EMT gives tumor cells increased motility and propensity to metastasize. As such, autophagy (striving for mere survival) and EMT (escaping from a hostile microenvironment) can be considered two opposite stress responses that are mutually exclusive [52]. Our observations of the coexistence of autophagy and EMT provoked by acute acidotic stress (see *S.A.* in Figs. 1f-g & S2c-S2d) clearly do not endorse this assumption. Rather, the results support a recent hypothesis that one of these two stress responses is a necessary requirement for the other, i.e., the EMT-related signaling pathways can either trigger or repress autophagy and that autophagy may provide energy for EMT or suppress EMT-induced metastatic spreading [52–54]. Further work is needed to clarify the molecular control mechanism and the crosstalk between acidic pH*e*-mediated autophagy and EMT, and to also determine whether disruption of this crosstalk can lead to dysfunctional responsiveness of tumor cells to the acidic pH*e* microenvironment.

Our findings in Fig. 1 indicate that the conventional statement of chronic autophagy induced by extracellular acidosis mainly served as an early stress-responsive survival mechanism, but not for later cellular adaptation to the prolonged acidic pH*e* exposure. Instead, solid tumors such as PDAC acquired alternative metabolic strategies to adapt to the constant and prolonged microenvironmental acidification (Figs. 1j-m, 2, & S2e-S2j). Therefore, the concept of targeting acidic pH*e*-induced autophagy as a therapeutic strategy for anticancer therapy may need to be adjusted [14, 15]. One would expect little or no autophagy in some, if not all, long-term acid-adapted tumor cells from cancer patients.

Increasing evidence has suggested that environmental stress alteration is associated with rapid adjustments of mitochondrial function often accompanied by aberrant mitochondrial ROS production and fusion/fission activity [46, 48]. The description is similar to our findings of the early acute effect of extracellular acidosis on PDAC cells causing severe mitochondrial damage coupled with reduced oxidative phosphorylation and ATP production (see *S.A.* vs *Ctrl* or *Buff*, Figs. 1j-k & S2e-S2f). These metabolic alterations were attributed to a rapid induction of massive SIMH to counter the microenvironmental stress by preserving damaged mitochondria from mitophagic degradation. However, after PDAC cells become chronically acclimated to the constant stress of extracellular acidity, the damaged mitochondrial network undergoes an unexpectedly slow and progressive ultrastructural transformation from the interconnected reticular SIMH to the disconnected fragmented configuration (see Fig. 2 & Videos S3, S4 and S5).

Although we attempted a thorough and extensive literature search, we could not find any similar report on such gradual and year-long mitochondrial network dynamics starting from global SIMH initiation as an early responsive machinery followed by gradual disintegration as a late adaptative mechanism to acidic stress (Fig. 2). The closest comparison we could find was a recent study of chronic kidney disease showing a slow and gradual change in mitochondrial dynamics from fusion to fission in remnant renal masses from day 2 to day 28 after 5/6 nephrectomy in rats [55]. Another example of the critical role of shifting the mitochondrial dynamic equilibrium was seen in *S. cerevisiae*, in that it employed a delicate balance of fusion and fission to adjust mitochondrial dynamics in a stationary growth phase to resist prolonged dehydration stress [56]. These examples, together with our observations in human cells, signify the importance of mitochondrial network dynamics in both early stress response and late adaptation to the changing microenvironment. Figure 3a-b shows that successful disruptions of this dynamic mitochondrial network severely hampered the adaptive capability of tumor cells to the microenvironmental acidification, thus suggesting the feasibility of this strategy for future clinical applications.

Apart from fusion and fission, mitochondrial subcellular distribution is also regarded as a pivotal modulator for tumor malignancy even though many fundamental aspects remained unexplored [49]. We postulate that the accumulated bioenergetically active mitochondria at or near the leading edge of the cell help provide a local source of energy for cell membrane dynamics, migration, and invasion. We present evidence showing the impact of acidic pH*e* stress to the increased mitochondrial trafficking and subcellular localization. Upon early exposure to extracellular acidosis, most mitochondria in PDAC cells were fused into interconnected reticular networks (see *S.A.* in Fig. 2a-g) and spread throughout the cytoplasm via anchoring to the actin filaments toward cell periphery (see *S.A.* in Fig. 4a-b). At this early acidosis-responsive *S.A.* state, PDAC cells with substantial mitochondrial trafficking acquired a more aggressive phenotype than the control groups at pH*e* 7.4 (see *S.A.* vs *Ctrl* or *Buff*, Fig. 4f-h, m & n). This phenomenon is somewhat unexpected, as tumor cells at this early stress-responsive stage are relatively fragile with a proliferation rate that was severely restrained and that most mitochondria are in hyperfused state associated with ATP insufficiency. However, many mitochondria were fragmented from the filamentous tubular network into spherical active organelles after exposure to acidic stress conditions for a sufficiently long period (see *L.A.* in Fig. 2a-g). They remained at the leading edge of the cell to support ATP-consuming activities for tumor cell motility and invasion (see *L.A.* in Figs. 1j-k & 4a-n). Quantification of the differential expression levels of a variety of metastasis-related molecules (Fig. 4k-l) confirmed that the acid-adapted PDAC cells progressively evolved into a more malignant state with increased migratory and invasive potential upon stimulation. More extensive studies on these protein molecules and associated pathways are needed to elucidate the molecular mechanism involved in PDAC early responses and late adaptation to extracellular acidification. These can determine whether the alteration of mitochondrial subcellular distributions may prevent accumulation of mitochondria at the cell periphery to subsequently impair PDAC motility and invasiveness under an acidic pH*e* microenvironment.

Tumor progression is an evolutionary and ecological process [11, 17, 57]. At the cellular selection level, only the cell population that is best adjusted to survive in a microenvironmental ecosystem will persist. For a cell population to persist, it must be able to evolve to maintain its fitness and adapt necessary phenotype and metabolic rewiring to be more successful in thriving and progressing within a new or changing microenvironment. However, Noë et al. recently stated that much of today’s discourse on PDAC fails to include an evolutionary perspective [57]. They suggested that PDAC cells should be viewed as evolving living organisms interacting with their microenvironment [57]. In this study, PDAC cells exhibited almost totally different responses and adaptations to the acute and chronic acidic microenvironmental pressure (Figs. 1, 2 and 3). In light of evolution, PDAC cells under extracellular acidosis constantly evolved toward a more aggressive phenotypic state in a time-dependent manner (Fig. 4). The identified enrichment of four functional hallmarks (advanced malignancy, EMT, migration & invasion) provides a strong and selective advantage for PDAC cells exposed to extended extracellular acidity; LKB1 and adherens_junctions_interactions canonical pathways are primarily involved in the metabolism and growth control of PDAC cells during early acidic pH*e* stress (Fig. 5a-b).

Next, comprehensive heatmap analyses uncovered thirteen acid-adaptation up-regulated and six down-regulated genes whose expression levels are respectively negatively and positively correlated with the overall survival of pancreatic cancer patients (Fig. 5c-i). To further classify the molecular targets specifically involved in the long-term adaptation of PDAC cells to the acidic microenvironment, we identified and annotated two distinct gene sets (the chronic_acidosis_adaptation up-regulated signature and the chronic_acidosis_adaptation down-regulated signature; see Tables S4 & S5). These expression levels are differentially impacted by prolonged acidic pH*e* exposure (Fig. 6). There were fourteen up-regulated genes and one down-regulated gene significantly associated with the overall survival of pancreatic cancer patients (Fig. 6b). Most of these genes were previously unidentified by short-term analyses of the impacts of extracellular acidosis on tumor cells. These fifteen acidic-pHe-related molecules—together with the nineteen genes identified from GSEA analysis in Fig. 5—are potential biomarkers for early diagnosis and therapeutic targets of PDAC. Deeper functional explorations of these acidosis-related genes are necessary to validate their roles in early response or late adaptation of pancreatic tumor cells to extracellular acidification. Finally, our findings fill a relevant knowledge gap in how solid tumor cells such as PDAC cells evolve to withstand, respond, reprogram, and ultimately adapt to the prolonged and persistent acidic pH*e* microenvironment.

## Conclusions

We present evidence here for distinct mitochondrial network dynamics characterized by rapid responsive capabilities followed by slow adaptive plasticity to the acidic tumor microenvironment. Successfully disruption of this dynamic mitochondrial network was shown to cause an inadequate response or intolerance to extracellular acidosis especially under acute exposure. A long-term acid-adapted cell population with significantly increased metastatic potentials was obtained; 34 molecular targets were found to be closely related to the overall pancreatic cancer patient survival. These were previously unable to be identified by short-term experiments for the effect of extracellular acidosis. These acidic-pH*e*-related molecules may be potential targets for the development of diagnosis and treatment against PDAC.

## Supplementary Information

**Additional file 1: Figure S1.** Time-course protein expression analysis in SUIT-2 PDAC cells exposed to extracellular acidity from day 0 to day 7. Whole cell lysates were extracted from representative acid pH*e*-treated SUIT-2 PDAC cells subjected to Western blotting using the indicated antibodies to determine protein expression levels of (**a**) pro-survival and autophagy markers; (**b**) mitochondrial dynamics regulators; and (**c**) metastasis-related molecules.

**Additional file 2: Figure S2**. Short-term response and long-term adaptation of BxPC-3 PDAC cells to extracellular acidotic stress. **a** The time effects of extracellular acidification on the proliferative responses of BxPC-3 PDAC cells were evaluated with MTT assay; data are shown as the mean ± SD (*n* = 3) and presented as fold-change relative to day 0. **b** Cell-cycle profiles in five cell states cultured under various control and acidic pH*e* conditions. The results are displayed as column graphs, and the error bars indicate means ± SD (*n =* 3). Asterisks represent significant differences between the indicated groups versus the *Buff* group. **c** Tumor cell morphological changes have a dynamic presence of autophagic vacuoles under acidic conditions. The bottom panels display enlargement of yellow dotted-boxed area from the respective top panels. Scale bar, 50 μm. **d** Acute extracellular acidification induced the formation of autophagic vacuoles co-localized with lysosomes. This autophagic flux became less apparent after tumor cells progressively adapt to acidic stress. Scale bar, 50 μm. **e-h** Seahorse assays of BxPC-3 PDAC cells upon different periods of acidic pH*e* exposure with oxygen consumption rates (OCR) and extracellular acidification rates (ECAR) displayed as mean ± SD (*n =* 4). FCCP: carbonyl cyanide-4(trifluoromethoxy)phenylhydrazone. **i-j** N-SIM images reveal a dynamic mitochondrial network in live BxPC-3 cells upon acute and chronic acidic exposures. Scale bar, 5 μm. **k** Enhanced degradation of DQ-collagen IV (green) in live BxPC-3 cells (red) under acidic pH*e*. Scale bar: 20 μm. **l** Western blots of representative cell cycle, autophagy, pro-survival, mitochondrial dynamics, and invasion-related markers. NS: not significant (*p* > 0.05); *p < 0.05; **p < 0.01; ***p < 0.001.

**Additional file 3: Figure S3.** Genome-wide expression analysis suggests a negligible buffer effect on PDAC cells under acidotic stress conditions. **a** Principal component analysis (PCA) of microarray-based genome-wide gene expression profiles of SUIT-2 PDAC cells subjected to various periods of normal or acidic culture conditions. Six different cell sets (each in duplicate) were analyzed: one control cell group (*Ctrl*) grown in control medium at pH*e* 7.4; two buffered groups (*Buff*) cultured in control medium supplemented with 25 mM HEPES/PIPES buffer at pH*e* 7.4 for either a short (2-3 weeks) or a long (10-12 months) period of time; and three acid-treated groups cultivated in control medium adjusted to pH*e* 6.7 with the addition of 25 mM HEPES/PIPES. The PCA plot displays the variances of 6 cell sets in terms of principal components and reveals the most significant of these on the x-, y-, and z-axis. The 1st, 2nd, and 3rd principal components cover 20.5, 15.2, and 10.5% of the total variances, respectively. **b** Of 67,528 gene probes analyzed on the *Ctrl* and *Buff* cell groups, more than 99% remained unchanged in terms of expression in PDAC cells treated with continuous Good’s zwitterionic buffers. After ~ 1 year of buffer culture and passage, 263 probes (0.39%) were found to be up-regulated (fold change ≥2), and 372 probes (0.55%) were down-regulated to the point of a significant difference (fold change ≤0.5). Note: *Tumor cells incubated with the addition of HEPES/PIPES buffer at pH*e* 7.4 for two weeks, a culture period similar to those in *S.A.* (short-term acidification) cell group. **Tumor cells incubated in HEPES/PIPES at pH*e* 7.4 for ~ 1 year—a culture period similar to those in *L.A.* (long-term acidification) cell group.

**Additional file 4: Table S1.** List of antibodies, suppliers, and working dilutions used in this study.

**Additional file 5: Table S2.** Sequences of small interfering RNAs used in this study.

**Additional file 6: Table S3.** IPA annotation of representative functional pathways associated with long-term adaptation of PDAC tumor cells to an acidic pH*e* microenvironment.

**Additional file 7: Table S4.** List of significantly up-regulated differentially expressed genes associated with long-term adaptation of PDAC tumor cells to an acidic pH*e* microenvironment.

**Additional file 8: Table S5.** List of significantly down-regulated and differentially expressed genes associated with long-term adaptation of PDAC tumor cells to acidic pH*e* microenvironment.

**Additional file 9: Video S1.** Representative N-SIM super-resolution video showing the mitochondria of the control (*Ctrl*) group of SUIT-2 PDAC cells at pH*e* 7.4

**Additional file 10: Video S2.** Representative N-SIM super-resolution video showing the mitochondria of the buffered (*Buff*) group of SUIT-2 PDAC cells at pH*e* 7.4.

**Additional file 11: Video S3.** Representative N-SIM super-resolution video showing the mitochondria of the *S.A. *group of SUIT-2 PDAC cells at pH*e* 6.7.

**Additional file 12: Video S4.** Representative N-SIM super-resolution video showing the mitochondria of the *M.A.* group of SUIT-2 PDAC cells at pH*e* 6.7.

**Additional file 13: Video S5.** Representative N-SIM super-resolution video showing the mitochondria of the *L.A.* group of SUIT-2 PDAC cells at pH*e* 6.7.

## Data Availability

Original microarray data used in this study were deposited in Gene Expression Omnibus (GEO) under accession number GSE180043. The authors declare that all the datasets supporting the conclusions of this article are included within the article and its additional files, or are available from the corresponding author upon reasonable request.

## Abbreviations

BSA: Bovine serum albumin
CDK: Cyclin-dependent kinase
DEGs: Differential expressed genes
DMSO: Dimethyl sulfoxide
ECAR: Extracellular acidification rate
EMT: Epithelial-mesenchymal transition
FBS: Fetal bovine serum
FCCP: Carbonyl cyanide-4(trifluoromethoxy)phenylhydrazone
GSEA: Gene set enrichment analysis
HEPES: N-(2-hydroxyethyl)piperazine-N′-(2-ethanesulfonic acid)
HRP: Horseradish peroxidase
MTT: Thiazolyl blue tetrazolium bromide
OCR: Oxygen consumption rate
PBS: Phosphate buffered saline
PCA: Principal component analysis
PDAC: Pancreatic ductal adenocarcinoma
PFA: Paraformaldehyde
pH*e*: Extracellular pH
pH*i*: Intracellular pH
PIPES: Piperazine-N,N′-bis (2-ethanesulfonic acid)
ROS: Reactive oxygen species
SIMH: Stress-induced mitochondrial hyperfusion
TEM: Transmission electron microscopy
TMRE: Tetramethylrhodamine ethyl ester
TVDT: Tumor volume doubling time
