# Supplementary figures and images for: Identification of distinct slow mode of reversible adaptation of pancreatic ductal adenocarcinoma to the prolonged acidic pH microenvironment
Source: J Exp Clin Cancer Res. 2022 Apr 11;41:137. doi: 10.1186/s13046-022-02329-x (PMC8996570; doi:10.1186/s13046-022-02329-x)

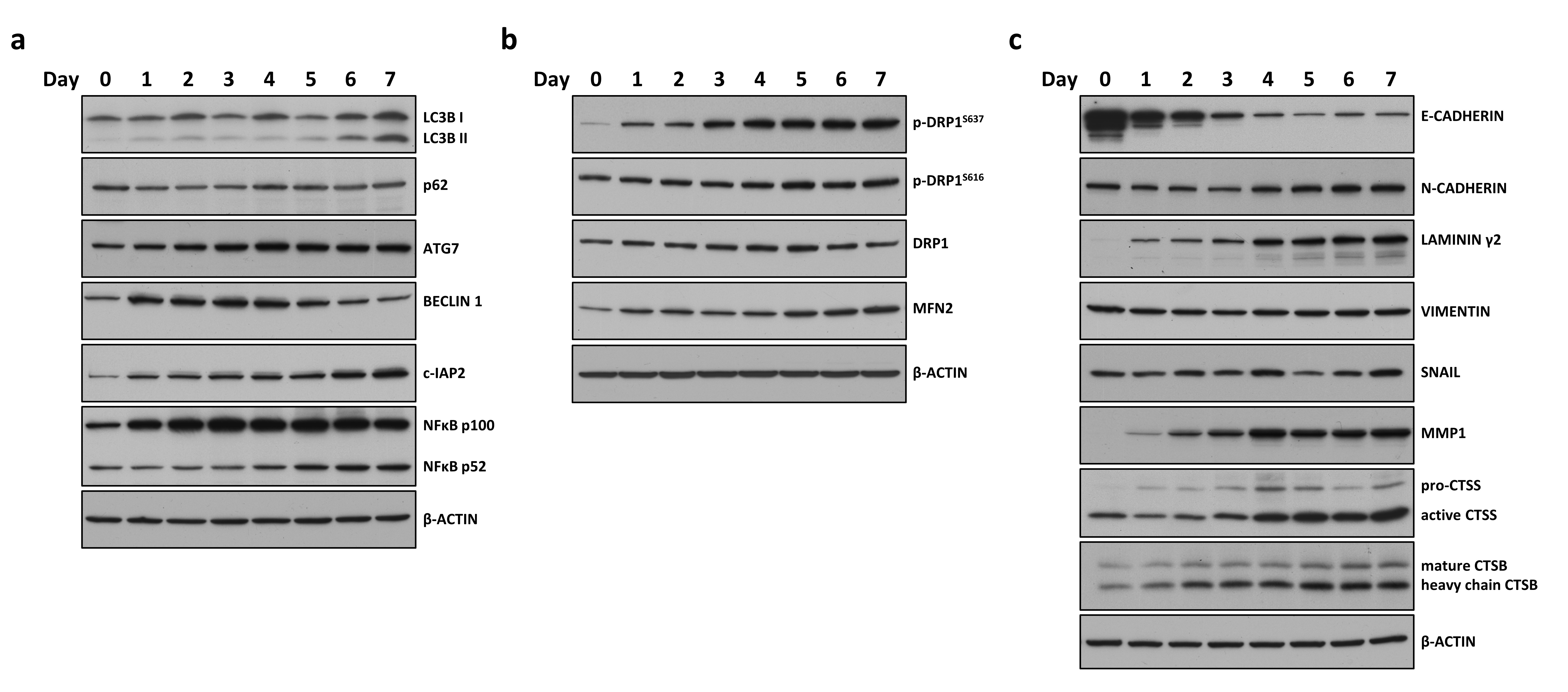

Supplement: Supplementary file 1 — Additional file 1: Figure S1. Time-course protein expression analysis in SUIT-2 PDAC cells exposed to extracellular acidity from day 0 to day 7. Whole cell lysates were extracted from representative acid pHe-treated SUIT-2 PDAC cells subjected to Western blotting using the indicated antibodies to determine protein expression levels of (a) pro-survival and autophagy markers; (b) mitochondrial dynamics regulators; and (c) metastasis-related molecules. [file 13046_2022_2329_MOESM1_ESM.jpg]

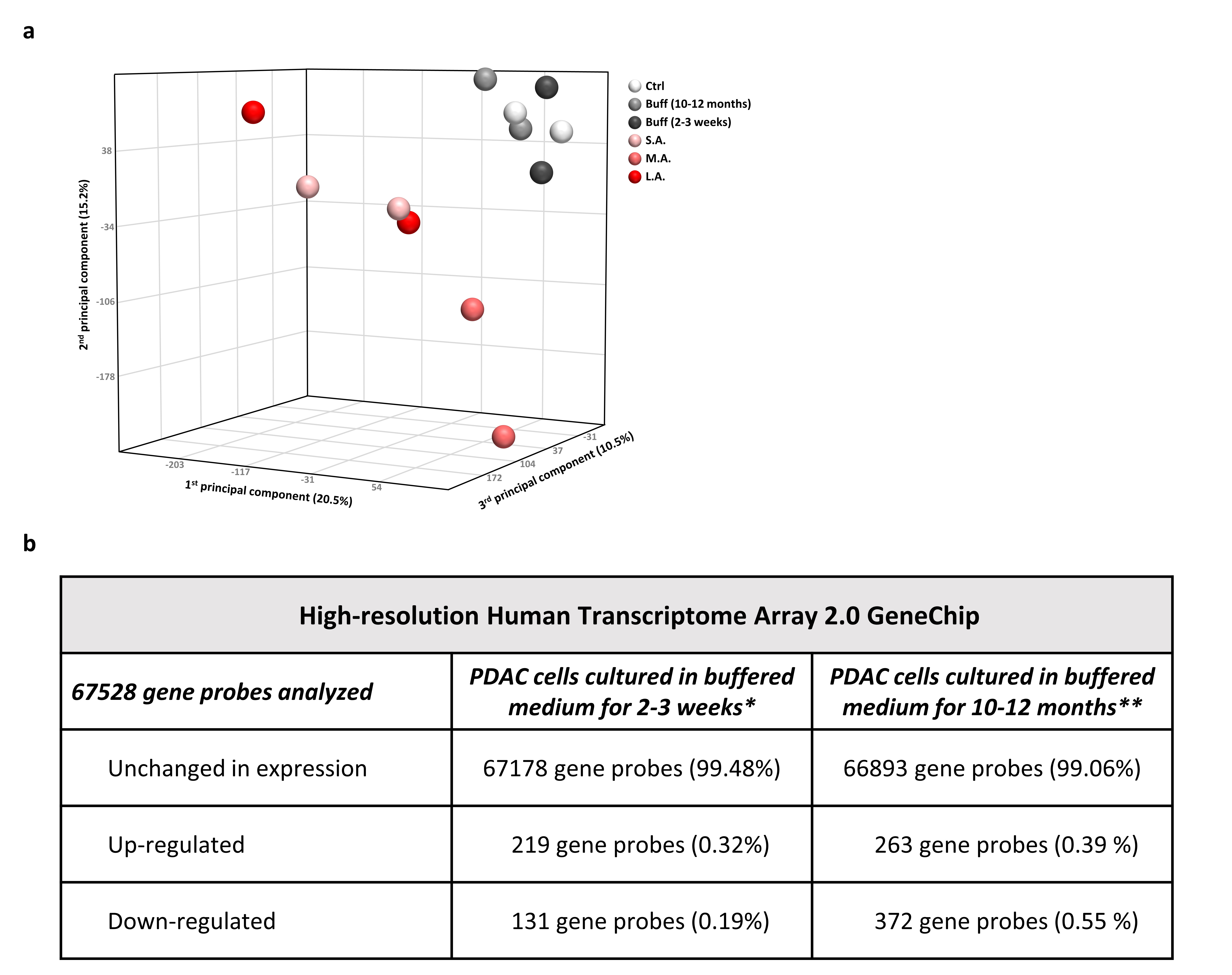

Supplement: Supplementary file 3 — Additional file 3: Figure S3. Genome-wide expression analysis suggests a negligible buffer effect on PDAC cells under acidotic stress conditions. a Principal component analysis (PCA) of microarray-based genome-wide gene expression profiles of SUIT-2 PDAC cells subjected to various periods of normal or acidic culture conditions. Six different cell sets (each in duplicate) were analyzed: one control cell group (Ctrl) grown in control medium at pHe 7.4; two buffered groups (Buff) cultured in control medium supplemented with 25 mM HEPES/PIPES buffer at pHe 7.4 for either a short (2-3 weeks) or a long (10-12 months) period of time; and three acid-treated groups cultivated in control medium adjusted to pHe 6.7 with the addition of 25 mM HEPES/PIPES. The PCA plot displays the variances of 6 cell sets in terms of principal components and reveals the most significant of these on the x-, y-, and z-axis. The 1st, 2nd, and 3rd principal components cover 20.5, 15.2, and 10.5% of the total variances, respectively. b Of 67,528 gene probes analyzed on the Ctrl and Buff cell groups, more than 99% remained unchanged in terms of expression in PDAC cells treated with continuous Good’s zwitterionic buffers. After ~ 1 year of buffer culture and passage, 263 probes (0.39%) were found to be up-regulated (fold change ≥2), and 372 probes (0.55%) were down-regulated to the point of a significant difference (fold change ≤0.5). Note: *Tumor cells incubated with the addition of HEPES/PIPES buffer at pHe 7.4 for two weeks, a culture period similar to those in S.A. (short-term acidification) cell group. **Tumor cells incubated in HEPES/PIPES at pHe 7.4 for ~ 1 year—a culture period similar to those in L.A. (long-term acidification) cell group. [file 13046_2022_2329_MOESM3_ESM.jpg]
