# Supplementary material for: Identification of distinct slow mode of reversible adaptation of pancreatic ductal adenocarcinoma to the prolonged acidic pH microenvironment
Source: J Exp Clin Cancer Res. 2022 Apr 11;41:137. doi: 10.1186/s13046-022-02329-x (PMC8996570; doi:10.1186/s13046-022-02329-x)
Supplement: Supplementary file 4 — Additional file 4: Table S1. List of antibodies, suppliers, and working dilutions used in this study. [file 13046_2022_2329_MOESM4_ESM.docx]

**Table S1. List of antibodies, suppliers, and working dilutions used in this study**^¶^

| **Primary Antibodies** | | | | | |
| --- | --- | --- | --- | --- | --- |
| **Antibody** | **Host** | **Type** | **Supplier** | **Catalog #** | **Dilution** |
| ACTB (β-actin) | Rabbit | Monoclonal | Cell Signaling | 4970 | 1:5000 |
| ACTR3/ARP3 (Actin-related protein 3) | Rabbit | Polyclonal | Cell Signaling | 4738 | 1:2000 |
| ARHGAP29 (Rho GTPase activating protein 29) | Rabbit | Polyclonal | Abcam | ab85853 | 1:2000 |
| ATG7 (Autophagy-related 7) | Rabbit | Monoclonal | Cell Signaling | 8558 | 1:4000 |
| BAK1/BAK (BCL2 antagonist/killer 1) | Rabbit | Monoclonal | Cell Signaling | 12105 | 1:4000 |
| BECN1 (Beclin-1) | Rabbit | Polyclonal | Abcam | ab51031 | 1:2000 |
| BIRC3/c-IAP2 (Baculoviral IAP repeat-containing 3) | Rabbit | Monoclonal | Cell Signaling | 3130 | 1:2000 |
| BNIP3 (BCL2 interacting protein 3) | Rabbit | Monoclonal | Cell Signaling | 44060 | 1:2000 |
| BNIP3L/NIX (BCL2 interacting protein 3-like) | Rabbit | Monoclonal | Cell Signaling | 12396 | 1:2000 |
| BAX (BCL2-associated X, apoptosis regulator) | Rabbit | Monoclonal | Cell Signaling | 5023 | 1:4000 |
| CCND1 (Cyclin D1) | Rabbit | Monoclonal | Cell Signaling | 2978 | 1:2000 |
| CCNE1 (Cyclin E1) | Mouse | Monoclonal | BD Biosciences | 551159 | 1:2000 |
| CDH1 (E-cadherin) | Rabbit | Monoclonal | Cell Signaling | 3195 | 1:5000 |
| CDH2 (N-cadherin) | Mouse | Monoclonal | BD Biosciences | 610920 | 1:1000 |
| CDK4 (Cyclin-dependent kinase 4) | Mouse | Monoclonal | Merck | MAB8879 | 1:2000 |
| CDK6 (Cyclin-dependent kinase 6) | Rabbit | Monoclonal | Cell Signaling | 13331 | 1:2000 |
| CDKN1A/p21*^CIP1^* (Cyclin-dependent kinase inhibitor 1A) | Rabbit | Monoclonal | Cell Signaling | 2947 | 1:2000 |
| CDKN1B/p27*^KIP1^* (Cyclin-dependent kinase inhibitor 1B) | Rabbit | Monoclonal | Cell Signaling | 3686 | 1:2000 |
| CFL1 (Cofilin 1) | Rabbit | Monoclonal | Cell Signaling | 5175 | 1:2000 |
| p-CFL1*^Ser3^/*p-COFILIN1*^Ser3^* | Rabbit | Monoclonal | Cell Signaling | 3313 | 1:2000 |
| Cleaved CASP3 (Cleaved caspase 3) | Rabbit | Monoclonal | Cell Signaling | 9664 | 1:2000 |
| CTSB (Cathepsin B) | Goat | Polyclonal | R&D Systems | AF953 | 1:2000 |
| CTSS (Cathepsin S) | Goat | Polyclonal | R&D Systems | AF1183 | 1:2000 |
| DNM1L/DRP1 (Dynamin-1-like) | Rabbit | Monoclonal | Cell Signaling | 8570 | 1:2000 |
| p-DNM1L*^Ser616^*/p-DRP1*^Ser616^* | Rabbit | Polyclonal | Cell Signaling | 3455 | 1:2000 |
| p-DNM1L*^Ser637^*/p-DRP1*^Ser637^* | Rabbit | Polyclonal | Cell Signaling | 4867 | 1:2000 |
| DSG2 (Desmoglein 2) | Mouse | Monoclonal | R&D Systems | MAB947 | 1:2000 |
| FN1 (Fibronectin 1) | Mouse | Monoclonal | R&D Systems | MAB1918 | 1:2000 |
| HA-tag (Human influenza hemagglutinin) | Mouse | Monoclonal | Cell Signaling | 2367 | 1:5000 |
| LAMC2/LAMININ γ2 (Laminin subunit γ-2) | Mouse | Monoclonal | Santa Cruz | sc-28330 | 1:1000 |
| MAP1LC3B/LC3B (Microtubule-associated protein 1 light chain 3β) | Rabbit | Monoclonal | Cell Signaling | 3868 | 1:5000 |
| MFN2 (Mitofusin 2) | Rabbit | Monoclonal | Cell Signaling | 9482 | 1:2000 |
| NFKB2/NFκB2 (Nuclear factor κB subunit 2) | Mouse | Monoclonal | R&D Systems | MAB28881 | 1:2000 |
| OPA1 (OPA1 mitochondrial dynamin-like GTPase) | Mouse | Monoclonal | BD Biosciences | 612607 | 1:2000 |
| RAC1-CDC42 (Rac family small GTPase 1 complexed with [cell division cycle 42](https://www.genenames.org/data/gene-symbol-report/#!/hgnc_id/HGNC:1736)) | Rabbit | Polyclonal | Cell Signaling | 4651 | 1:2000 |
| p-RAC1-CDC42*^Ser71^* | Rabbit | Polyclonal | Cell Signaling | 2461 | 1:2000 |
| PFN1 (Profilin 1) | Rabbit | Monoclonal | Cell Signaling | 3246 | 1:2000 |
| SNAI1/SNAIL (Snail family transcriptional repressor 1) | Rabbit | Monoclonal | Cell Signaling | 3879 | 1:2000 |
| SQSTM1/p62 (Sequestosome 1) | Mouse | Monoclonal | Abcam | ab56416 | 1:5000 |
| ST3GAL1 (ST3 β-galactoside α-2,3-sialyltransferase 1) | Sheep | Polyclonal | R&D Systems | AF6905 | 1:2000 |
| VASP (Vasodilator-stimulated phosphoprotein) | Rabbit | Monoclonal | Invitrogen | MA5-14982 | 1:2000 |
| p-VASP*^Ser157^* | Rabbit | Polyclonal | Cell Signaling | 3111 | 1:2000 |
| VDAC1 (Voltage-dependent anion channel 1) | Rabbit | Polyclonal | Cell Signaling | 4866 | 1:2000 |
| VIM (Vimentin) | Rabbit | Monoclonal | Cell Signaling | 3390 | 1:2000 |
| WASF2/WAVE-2 (WASP family member 2) | Rabbit | Monoclonal | Cell Signaling | 3659 | 1:2000 |
| WASL/N-WASP (WASP-like actin nucleation promoting factor) | Rabbit | Monoclonal | Cell Signaling | 4848 | 1:2000 |
| **Secondary Antibodies** | | | | | |
| **Antibody** | **Host** | **Type** | **Supplier** | **Catalog #** | **Dilution** |
| HRP-linked anti-rabbit IgG | Goat | Polyclonal | Cell Signaling | 7074 | 1:10000 |
| HRP-linked anti-mouse IgG | Horse | Polyclonal | Cell Signaling | 7076 | 1:10000 |
| HRP-conjugated anti-sheep IgG | Donkey | Polyclonal | R&D Systems | HAF016 | 1:10000 |
| HRP-conjugated anti-goat IgG | Rabbit | Polyclonal | Merck | AP106P | 1:10000 |
| Alexa Fluor 488-conjugated anti-rabbit IgG | Donkey | Polyclonal | Thermo Fisher | A-21206 | 1:1000 |

*^¶^Gene symbols and alias names were assigned based on standard nomenclature as per the Human Genome Organization (HUGO) Nomenclature Committee (http://www.genenames.org).*
