# Supplementary material for: Identification of distinct slow mode of reversible adaptation of pancreatic ductal adenocarcinoma to the prolonged acidic pH microenvironment
Source: J Exp Clin Cancer Res. 2022 Apr 11;41:137. doi: 10.1186/s13046-022-02329-x (PMC8996570; doi:10.1186/s13046-022-02329-x)
Supplement: Supplementary file 5 — Additional file 5: Table S2. Sequences of small interfering RNAs used in this study. [file 13046_2022_2329_MOESM5_ESM.docx]

**Table S2. Sequences of small interfering RNAs used in this study**^¶^

| Target | Product | Catalog # | Target sequence (5’→3’) |  |
| --- | --- | --- | --- | --- |
|  |  |  |  | |
| MFN2 (Mitofusin 2) | ON-TARGET*plus* | L-012961-00-0005 | GACUAUAAGCUGCGAAUUA | |
|  | Human MFN2 (Gene id: 9927) |  | CAUGAGGCCUUUCUCCUUA | |
|  | siRNA SMARTpool |  | GCAACUCUAUCGUCACAGU | |
|  |  |  | GGUGGACGAUUACCAGAUG | |
|  |  |  |  | |
| Negative Control | ON-TARGET*plus* | D-001810-10-05 | UGGUUUACAUGUCGACUAA | |
|  | Non-targeting Control Pool |  | UGGUUUACAUGUUGUGUGA | |
|  |  |  | UGGUUUACAUGUUUUCUGA | |
|  |  |  | UGGUUUACAUGUUUUCCUA | |
|  |  |  |  | |

*^¶^All siRNAs were purchased from Dharmacon, Horizon Discovery, Lafayette, CO, USA.*
