# Supplementary material for: Identification of distinct slow mode of reversible adaptation of pancreatic ductal adenocarcinoma to the prolonged acidic pH microenvironment
Source: J Exp Clin Cancer Res. 2022 Apr 11;41:137. doi: 10.1186/s13046-022-02329-x (PMC8996570; doi:10.1186/s13046-022-02329-x)
Supplement: Supplementary file 6 — Additional file 6: Table S3. IPA annotation of representative functional pathways associated with long-term adaptation of PDAC tumor cells to an acidic pHe microenvironment. [file 13046_2022_2329_MOESM6_ESM.docx]

**Table S3.** **IPA annotation of representative functional pathways associated with long-term adaptation of PDAC tumor cells to an acidic pH*e* microenvironment^¶^**

| IPA function annotation | No. of  molecules | List of molecules |
| --- | --- | --- |
| *Advanced_malignant_tumor* | *146* | *ABCA1, ADRB2, AKR1C1/AKR1C2, AMER1, ANXA8/ANXA8L1, ARL4C, ATM, BCL10, BCR, BRCA1, C1R, C3, CALD1, CARD11, CASP8, CBL, CCND1, CCND2, CDCA7L, CDK6, CDKN2C, CEMIP, CHD1, CHEK1, CMTM8, CRYBG1, CXCL8, DAB2, DHTKD1, DNAJB6, DPP4, DPYD, DRD1, DUSP4, EFEMP1, ENG, EPB41L4B, EPHA2, EPHB6, ERCC1, ETV1, F3, FAM171A1, FGF18, FGF2, FGFR1, FLNA, FN1, FRY, FSTL3, FZD3, GLUL, GNA12, GNAS, GSTM3, GSTP1, H2AFX, HIF1A, HIST1H1C, HIST1H3B, HMGA1, HRH1, IDH2, IDS, IGF2, IL2RG, IL6, INSR, ITPR3, LAMA3, LCN2, LET-7, LGALS3, LGALS4, LGR4, LRIG1, LRRFIP2, LTA, LTB, MAP3K13, MIR-10, MIR-130, MIR-139, MIR-148, MIR-154, MIR-181, MIR-21, MIR-218, MIR-29, MIR-31, MIR-329, MIR-378, MIR-450, MIR-548, MIR-550, MIR-8, MITF, MLPH, MME, MMP1, MMP14, MMP9, MSX1, NEDD9, NEUROD1, NR3C1, NRAS, P2RY1, PDGFRB, PGF, PIK3CD, POLE3, PPP2R2C, PPP3CB, PTGS1, RAB31, RARB, RARG, RASA1, RGCC, RGS4, RRAS, SCN5A, SEL1L3, SEMA3C, SERPINE1, SERPINE2, SERPING1, SLC16A3, SLPI, SORT1, SPP1, STAG2, STAT3, TGFBR3, TOP2A, TP53, TRAF4, TUBG1, TUBG2, TXNIP, VCAM1, XRCC1, YES1, ZFP36L2, ZNF138* |
| *Cell_movement_of_tumor_cell_lines* | *193* | *ABCA1, ACSL4, ADAM10, ADRB2, AJAP1, ALCAM, ARL4C, B4GAT1, BCL10, BCR, BMP6, BRCA1, C3, CALD1, CASP8, CBL, CCL7, CCND1, CD40, CD82, CD99, CDCA7L, CDK14, CLCN4, CMTM8, CREB3, CTNNBIP1, CXCL8, CYP2J2, DAB2, DCN, DDB2, DNAJB4, DNAJB6, DOCK1, DOCK4, DPAGT1, DPP4, DRAM1, DSE, DSP, EFEMP1, EGR1, EIF4EBP1, ENG, EPHA2, EPHB6, EPS8, EREG, ETV4, EYA3, F2RL1, F3, FAT1, FGF2, FGFR1, FLNA, FLNC, FN1, FOSB, FSCN1, GAB1, GIT1, GMFG, GNA12, GNAI3, GNAS, GRB7, GRN, HIF1A, HMGA1, HOMER3, HSPA1A/HSPA1B, ID1, IGF2, IGFBP1, IGFBP3, IL18, IL6, IL6R, ILK, INPPL1, IPO7, IQGAP1, ITGA5, ITGA6, ITGBL1, LAMA3, LCN2, LCP2, LET-7, LGALS3, LGALS3BP, LIFR, LIMA1, LMCD1, LMO7, LPAR3, LRIG1, MALT1, MAP4K4, MAPRE3, MARCKS, MIR-10, MIR-130, MIR-1301, MIR-139, MIR-144, MIR-148, MIR-181, MIR-204, MIR-21, MIR-218, MIR-31, MIR-8, MIR100HG, MITF, MME, MMP1, MMP14, MMP9, MSX2, MYC, NEDD4, NEDD9, NET1, NEUROD1, NOTCH2, NR5A2, NRAS, P2RY2, PALLD, PGF, PIK3CD, PIK3R1, PLCG1, PLXNA1, POU5F1, PPARA, PPARG, PRKCA, PRKCD, PRKD1, PRKG1, PROM1, PTN, PVR, PXN, RAD9A, RAP1A, RAPGEF3, RARG, RASA1, RELA, RIOK3, ROBO1, S100A9, S1PR3, SCN5A, SDC2, SDC4, SEMA3A, SEMA3C, SERPINB5, SERPINE1, SERPINF1, SH2B3, SLC48A1, SLC9A3R2, SPARC, SPP1, SPSB1, SPTLC1, SSH1, STAP2, STAT3, STMN3, SUZ12, TAGLN2, TAZ, TBXAS1, TCF7L2, TGFB1I1, TGFBR3, THBS2, TMPO, TNC, TP53, TP53INP1, TP53INP2, TRAF4, USP33, VCAM1* |
| *Invasion_of_tumor_cell_lines* | *165* | *ABLIM1, ACSL4, ADAM10, ADM, ADRB2, AJAP1, ALCAM, ARHGAP24, AZGP1, B4GAT1, BCL10, BMP6, BRCA1, BTC, CBL, CCND1, CD82, CD99, CDK14, CDK5RAP3, CHD1, CMTM8, CTNNBIP1, CXCL8, CYP2J2, DAB2, DCN, DDB2, DNAJB4, DNAJB6, DNMT3B, DOCK1, DOCK4, DPP4, DRAM1, DSE, DSP, DUSP1, ECE1, EFEMP1, EGR1, EIF4EBP1, EPAS1, EPHA2, EPHB6, EPS8, EREG, ESM1, ETS2, ETV1, ETV4, EYA3, FAM129B, FAT1, FGF2, FGFR1, FN1, FSCN1, GIT1, GNA12, GNAI3, GNAL, GNAS, GRB7, GRN, HEXIM1, HIF1A, HIF1A-AS2, HMG20A, HMGA1, HSPA1A/HSPA1B, ID1, IGF2, IL6, ILK, INHBB, IPO7, IQGAP1, IRS2, ITGA5, JAM3, LCN2, LET-7, LGALS3, LGR4, LIMA1, LOXL2, LRIG1, MALT1, MAP2K6, MAP4K4, MARCKS, MIR-10, MIR-139, MIR-144, MIR-181, MIR-204, MIR-21, MIR-218, MIR-29, MIR-31, MIR-663, MIR-8, MITF, MMP1, MMP14, MMP9, MSX2, MYC, MYO9A, NEDD4, NEDD9, NET1, NOTCH2, PALLD, PIK3R1, PLCG1, PPARG, PRKCA, PRKCD, PXN, RAC3, RAD9A, RAP1A, RARG, RASA1, RELA, RIOK3, RND3, S100A9, S1PR3, SCN5A, SCUBE3, SDC2, SDC4, SEC24D, SERPINB5, SERPINE2, SERPINF1, SIPA1, SLC48A1, SLFN5, SOX2, SPARC, SPP1, SPSB1, ST6GALNAC5, STAT3, STK38L, SUZ12, TAGLN, TAGLN2, TCIRG1, TGFBR3, TMPO, TNC, TP53, TP53INP2, TRIM33, UBD, VANGL1, VCAM1, VRK2, WNT5B, ZNF350* |

*^¶^IPA (Ingenuity Pathway Analysis, Qiagen, Hilden, Germany) functional enrichment analysis of microarray data of SUIT-2 pancreatic tumor cells exposed to various periods of acidic pHe. Three representative functional categories were subjected to GSEA (Gene Set Enrichment Analysis, https://www.gsea-msigdb.org/gsea/index.jsp), with most of their gene molecules shown to be significantly activated upon chronic cellular adaptation to extracellular acidic stress.*
