# Supplementary material for: Identification of distinct slow mode of reversible adaptation of pancreatic ductal adenocarcinoma to the prolonged acidic pH microenvironment
Source: J Exp Clin Cancer Res. 2022 Apr 11;41:137. doi: 10.1186/s13046-022-02329-x (PMC8996570; doi:10.1186/s13046-022-02329-x)
Supplement: Supplementary file 7 — Additional file 7: Table S4. List of significantly up-regulated differentially expressed genes associated with long-term adaptation of PDAC tumor cells to an acidic pHe microenvironment. [file 13046_2022_2329_MOESM7_ESM.docx]

**Table S4.** **List of significantly up-regulated differentially expressed genes associated with long-term adaptation of PDAC tumor cells to an acidic pH*e* microenvironment^¶^**

| Gene Symbol | Description | Log_2_FC | *P*-value |
| --- | --- | --- | --- |
| OTTHUMG00000021149 | OTTHUMG00000021149 | 5.515 | 9.04E-04 |
| OTTHUMG00000014989 | OTTHUMG00000014989 | 4.621 | 1.37E-03 |
| CYP24A1 | Cytochrome P450 family 24 subfamily A member 1 | 4.522 | 3.03E-04 |
| OTTHUMG00000161178 | OTTHUMG00000161178 | 3.792 | 7.66E-04 |
| CLDN16 | Claudin 16 | 3.169 | 4.50E-04 |
| LINC00707 | LINC00707 | 3.092 | 2.05E-02 |
| DEPDC7 | DEP domain containing 7 | 3.018 | 9.92E-03 |
| LOC152225 | LOC152225 | 3.016 | 6.90E-03 |
| LOX | Lysyl oxidase | 2.813 | 2.70E-02 |
| PSG1 | Pregnancy specific β-1-glycoprotein 1 | 2.692 | 3.79E-02 |
| CDK14 | Cyclin dependent kinase 14 | 2.629 | 7.21E-04 |
| CTSC | Cathepsin C | 2.617 | 5.08E-03 |
| SLC6A15 | Solute carrier family 6 member 15 | 2.571 | 2.46E-04 |
| NTN4 | Netrin 4 | 2.506 | 7.99E-03 |
| EPAS1 | Endothelial PAS domain protein 1 | 2.213 | 5.60E-03 |
| BAI3 | Adhesion G protein-coupled receptor B3 | 2.205 | 1.48E-02 |
| IL7R | Interleukin 7 receptor | 2.203 | 3.59E-02 |
| POGLUT2 | Protein O-glucosyltransferase 2 | 2.155 | 3.50E-03 |
| NFKBIZ | NFκB inhibitor ζ | 2.055 | 3.83E-02 |
| CA2 | Carbonic anhydrase 2 | 1.978 | 4.05E-03 |
| SFTA1P | Surfactant associated 1, lncRNA | 1.951 | 1.44E-03 |
| ACSL4 | acyl-CoA synthetase long chain family member 4 | 1.928 | 4.66E-02 |
| HLA-DMB | Major histocompatibility complex, class II, DM β | 1.833 | 4.07E-02 |
| HIF1A | Hypoxia inducible factor 1 subunit α | 1.822 | 4.99E-02 |
| FBLN5 | Fibulin 5 | 1.782 | 1.84E-03 |
| OTTHUMG00000154512 | OTTHUMG00000154512 | 1.777 | 2.04E-02 |
| SNAPC1 | Small nuclear RNA activating complex polypeptide 1 | 1.773 | 1.27E-02 |
| THBS1 | Thrombospondin 1 | 1.757 | 1.51E-02 |
| CCDC80 | Coiled-coil domain containing 80 | 1.756 | 2.08E-02 |
| LPP | LIM domain containing preferred translocation partner in lipoma | 1.712 | 2.19E-03 |
| PSG5 | Pregnancy specific β-1-glycoprotein 5 | 1.690 | 1.45E-02 |
| MIR4668 | MicroRNA 4668 | 1.676 | 2.21E-02 |
| LOC729987 | LOC729987 | 1.585 | 1.21E-02 |
| MSMO1 | Methylsterol monooxygenase 1 | 1.577 | 2.31E-02 |
| OTTHUMG00000159250 | OTTHUMG00000159250 | 1.558 | 1.70E-02 |
| LOC100507487 | LOC100507487 | 1.525 | 1.83E-02 |
| SERPING1 | Serpin family G member 1 | 1.514 | 5.54E-04 |
| OTTHUMG00000015645 | OTTHUMG00000015645 | 1.502 | 4.00E-04 |
| FOSL2 | FOS like 2, AP-1 transcription factor subunit | 1.497 | 3.07E-02 |
| LOC654342 | LOC654342 | 1.483 | 2.98E-03 |
| MT1A | Metallothionein 1A | 1.476 | 6.01E-03 |
| MALT1 | MALT1 paracaspase | 1.466 | 2.55E-03 |
| ANKRD13A | Ankyrin repeat domain 13A | 1.435 | 2.26E-04 |
| TC2N | Tandem C2 domains, nuclear | 1.425 | 4.25E-02 |
| LGR4 | Leucine rich repeat containing G protein-coupled receptor 4 | 1.410 | 1.45E-02 |
| PALLD | Palladin, cytoskeletal associated protein | 1.396 | 6.96E-03 |
| FLNC | Filamin C | 1.385 | 3.72E-03 |
| MT1F | Metallothionein 1F | 1.369 | 2.06E-02 |
| ZPLD1 | Zona pellucida like domain containing 1 | 1.362 | 1.31E-02 |
| PRR16 | Proline rich 16 | 1.350 | 3.49E-03 |
| SLIT2 | Slit guidance ligand 2 | 1.343 | 4.69E-02 |
| EFEMP1 | EGF containing fibulin extracellular matrix protein 1 | 1.299 | 3.04E-03 |
| STRIP2 | Striatin interacting protein 2 | 1.295 | 2.78E-02 |
| MIR99AHG | Mir-99a-let-7c cluster host gene | 1.294 | 4.35E-02 |
| ST3GAL1 | ST3 β-galactoside α-2,3-sialyltransferase 1 | 1.293 | 3.37E-02 |
| INSIG1 | Insulin induced gene 1 | 1.280 | 4.13E-02 |
| LIMCH1 | LIM and calponin homology domains 1 | 1.270 | 1.28E-02 |
| ECSCR | Endothelial cell surface expressed chemotaxis/apoptosis regulator | 1.259 | 3.07E-02 |
| OSMR | Oncostatin M receptor | 1.247 | 2.40E-02 |
| TFPI | Tissue factor pathway inhibitor | 1.237 | 9.60E-03 |
| MT1M | Metallothionein 1M | 1.233 | 4.51E-04 |
| MIR3689C | MicroRNA 3689c | 1.219 | 8.52E-03 |
| NLGN1 | Neuroligin 1 | 1.210 | 3.22E-04 |
| OTTHUMG00000157590 | OTTHUMG00000157590 | 1.188 | 4.57E-04 |
| OLFML2B | Olfactomedin like 2B | 1.187 | 3.91E-03 |
| KCNT2 | Potassium sodium-activated channel subfamily T member 2 | 1.184 | 8.71E-03 |
| MIR3689A | MicroRNA 3689a | 1.180 | 2.04E-02 |
| OR5V1 | Olfactory receptor family 5 subfamily V member 1 | 1.173 | 4.68E-03 |
| PRRG4 | Proline rich and Gla domain 4 | 1.168 | 3.09E-03 |
| GSTM4 | Glutathione S-transferase mu 4 | 1.151 | 3.19E-02 |
| DRAM2 | DNA damage regulated autophagy modulator 2 | 1.149 | 3.27E-02 |
| MMP19 | Matrix metallopeptidase 19 | 1.146 | 6.71E-03 |
| ZBTB38 | Zinc finger and BTB domain containing 38 | 1.139 | 9.47E-04 |
| LOC645166 | LOC645166 | 1.108 | 8.28E-03 |
| ST6GALNAC5 | ST6 N-acetylgalactosaminide α-2,6-sialyltransferase 5 | 1.085 | 2.26E-04 |
| VWDE | von Willebrand factor D and EGF domains | 1.070 | 6.34E-03 |
| MRPL33 | Mitochondrial ribosomal protein L33 | 1.068 | 1.31E-05 |
| PTN | Pleiotrophin | 1.068 | 5.39E-03 |
| RNA5SP296 | RNA, 5S ribosomal pseudogene 296 | 1.053 | 1.75E-02 |
| P3H2 | Prolyl 3-hydroxylase 2 | 1.047 | 8.38E-04 |
| NRAS | NRAS proto-oncogene, GTPase | 1.045 | 2.27E-04 |
| CLCC1 | Chloride channel CLIC like 1 | 1.037 | 2.10E-05 |
| FAM117B | Family with sequence similarity 117 member B | 1.037 | 1.88E-03 |
| BABAM2-AS1 | BABAM2 antisense RNA 1 | 1.028 | 2.32E-02 |
| OTTHUMG00000012023 | OTTHUMG00000012023 | 1.020 | 2.15E-02 |
| MAGI2-AS1 | MAGI2 antisense RNA 1 | 1.017 | 4.38E-03 |
| LOC401321 | LOC401321 | 1.015 | 1.41E-02 |
| CFAP299 | Cilia and flagella associated protein 299 | 1.010 | 1.83E-02 |
| PTGFRN | Prostaglandin F2 receptor inhibitor | 1.008 | 1.85E-02 |
| EPB41 | Erythrocyte membrane protein band 4.1 | 1.007 | 1.45E-03 |
| CTTNBP2NL | CTTNBP2 N-terminal like | 1.005 | 1.46E-03 |
| ANO2 | Anoctamin 2 | 1.004 | 3.40E-03 |
| OTTHUMG00000035229 | OTTHUMG00000035229 | 1.003 | 1.66E-02 |
| PAM | Peptidylglycine α-amidating monooxygenase | 1.001 | 7.52E-03 |

*^¶^Genes differentially expressed in SUIT-2 PDAC tumor cells exposed to long-term extracellular acidity (L.A. group) were determined by Affymetrix GeneChip Human Transcriptome Array 2.0, and compared with gene expression profiles of cells treated with acute short-term acidification (S.A. group). A total of 94 significantly up-regulated genes (Log_2_FC ≥ 1, p-value < 0.05) were identified in the L.A. cell group as compared to those in the S.A. cell group. The very few genes up- or down-regulated found in SUIT-2 tumor cells exposed to HEPES/PIPES buffer were excluded for the purpose of paired analyses (see comparison between Ctrl and Buff cell groups).*
