# Supplementary material for: Identification of distinct slow mode of reversible adaptation of pancreatic ductal adenocarcinoma to the prolonged acidic pH microenvironment
Source: J Exp Clin Cancer Res. 2022 Apr 11;41:137. doi: 10.1186/s13046-022-02329-x (PMC8996570; doi:10.1186/s13046-022-02329-x)
Supplement: Supplementary file 8 — Additional file 8: Table S5. List of significantly down-regulated and differentially expressed genes associated with long-term adaptation of PDAC tumor cells to acidic pHe microenvironment. [file 13046_2022_2329_MOESM8_ESM.docx]

**Table S5. List of significantly down-regulated and differentially expressed genes associated with long-term adaptation of PDAC tumor cells to acidic pH*e* microenvironment^¶^**

| Gene Symbol | Description | Log_2_FC | P-value |
| --- | --- | --- | --- |
| CST1 | Cystatin SN | -7.687 | 7.36E-03 |
| ADGRF1 | Adhesion G protein-coupled receptor F1 | -4.458 | 1.95E-04 |
| VCAN | Versican | -4.234 | 2.44E-02 |
| COL6A3 | Collagen type VI α 3 chain | -4.081 | 1.40E-03 |
| CALB2 | Calbindin 2 | -3.774 | 3.64E-03 |
| ANKRD1 | Ankyrin repeat domain 1 | -3.306 | 3.30E-02 |
| SORL1 | Sortilin-related receptor 1 | -3.067 | 1.94E-03 |
| ITGA6 | Integrin subunit α 6 | -2.831 | 2.23E-06 |
| SLC9A3R2 | SLC9A3 regulator 2 | -2.803 | 4.79E-04 |
| CNIH3-AS2 | CNIH3 antisense RNA 2 | -2.723 | 2.00E-04 |
| WNT7A | Wnt family member 7A | -2.723 | 7.86E-03 |
| IFI44L | Interferon induced protein 44 like | -2.669 | 4.56E-02 |
| MMP1 | Matrix metallopeptidase 1 | -2.666 | 2.45E-02 |
| SULF2 | Sulfatase 2 | -2.645 | 1.06E-02 |
| ROR1 | Receptor tyrosine kinase like orphan receptor 1 | -2.434 | 4.49E-03 |
| LTB | Lymphotoxin β | -2.386 | 4.99E-02 |
| LOC101060026 | LOC101060026 | -2.385 | 5.75E-03 |
| MYO1D | Myosin ID | -2.301 | 8.43E-03 |
| F2RL1 | F2R like trypsin receptor 1 | -2.286 | 2.22E-03 |
| LOC101059935 | LOC101059935 | -2.212 | 5.44E-03 |
| TESC | tescalcin | -2.157 | 4.90E-02 |
| VCAN-AS1 | VCAN antisense RNA 1 | -2.124 | 4.77E-02 |
| RNA5SP348 | RNA, 5S ribosomal pseudogene 348 | -2.028 | 2.34E-03 |
| ITGA2 | Integrin subunit α 2 | -1.996 | 4.28E-02 |
| ANKRD20A1 | Ankyrin repeat domain 20 family member A1 | -1.987 | 5.22E-03 |
| ANKRD20A2P | Ankyrin repeat domain 20 family member A2, pseudogene | -1.987 | 5.56E-03 |
| NRIP1 | Nuclear receptor interacting protein 1 | -1.979 | 1.88E-03 |
| KLHL4 | Kelch like family member 4 | -1.967 | 2.42E-03 |
| EFNB2 | Ephrin B2 | -1.966 | 1.91E-02 |
| PLEK2 | Pleckstrin 2 | -1.937 | 9.83E-05 |
| GMFG | Glia maturation factor γ | -1.922 | 3.05E-02 |
| ANKRD20A3P | Ankyrin repeat domain 20 family member A3, pseudogene | -1.909 | 8.17E-03 |
| ANKRD20A4P | Ankyrin repeat domain 20 family member A4, pseudogene | -1.898 | 9.95E-03 |
| VCAM1 | Vascular cell adhesion molecule 1 | -1.865 | 1.15E-02 |
| OAS3 | 2'-5'-oligoadenylate synthetase 3 | -1.857 | 3.38E-02 |
| NET1 | Neuroepithelial cell transforming 1 | -1.833 | 2.96E-03 |
| TINAGL1 | Tubulointerstitial nephritis antigen like 1 | -1.821 | 1.84E-03 |
| VTRNA2-1 | Vault RNA 2-1 | -1.808 | 4.91E-03 |
| FOSL1 | FOS like 1, AP-1 transcription factor subunit | -1.738 | 2.08E-02 |
| MIR1184-2 | MicroRNA 1184-2 | -1.730 | 7.37E-03 |
| MIR1184-1 | MicroRNA 1184-1 | -1.730 | 7.37E-03 |
| B3GALT5-AS1 | B3GALT5 antisense RNA 1 | -1.720 | 4.97E-03 |
| GRHL2 | Grainyhead like transcription factor 2 | -1.716 | 4.51E-02 |
| RNA5SP325 | RNA, 5S ribosomal pseudogene 325 | -1.694 | 4.40E-03 |
| TFAP2C | Transcription factor AP-2 γ | -1.690 | 2.11E-02 |
| C11orf1 | Chromosome 11 open reading frame 1 | -1.690 | 3.20E-05 |
| RESF1 | Retroelement silencing factor 1 | -1.678 | 2.05E-04 |
| RNA5SP243 | RNA, 5S ribosomal pseudogene 243 | -1.672 | 5.11E-03 |
| SNTB1 | Syntrophin β 1 | -1.658 | 3.49E-02 |
| MIRLET7A2 | MicroRNA let-7a-2 | -1.637 | 3.52E-02 |
| SNORD14E | Small nucleolar RNA, C/D box 14E | -1.636 | 3.84E-03 |
| JAG1 | Jagged canonical Notch ligand 1 | -1.628 | 3.49E-05 |
| KCNN4 | Potassium calcium-activated channel subfamily N member 4 | -1.624 | 1.67E-02 |
| PLAAT3 | Phospholipase A and acyltransferase 3 | -1.614 | 1.95E-02 |
| RNU5D-2P | RNA, U5D small nuclear 2, pseudogene | -1.589 | 2.54E-02 |
| IFI35 | Interferon induced protein 35 | -1.589 | 1.15E-02 |
| MYEOV | Myeloma overexpressed | -1.586 | 3.57E-02 |
| FA2H | Fatty acid 2-hydroxylase | -1.573 | 6.57E-03 |
| SNORA59B | Small nucleolar RNA, H/ACA box 59B | -1.571 | 3.26E-02 |
| RNA5SP85 | RNA, 5S ribosomal pseudogene 85 | -1.555 | 1.58E-03 |
| LHX1 | LIM homeobox 1 | -1.553 | 2.29E-03 |
| KLF12 | Kruppel like factor 12 | -1.552 | 1.26E-04 |
| SPNS2 | Sphingolipid transporter 2 | -1.538 | 1.78E-02 |
| RNU5D-1 | RNA, U5D small nuclear 1 | -1.538 | 7.55E-03 |
| SNORA2B | Small nucleolar RNA, H/ACA box 2B | -1.534 | 7.40E-03 |
| RASEF | RAS and EF-hand domain containing | -1.531 | 3.69E-03 |
| RNA5SP199 | RNA, 5S ribosomal pseudogene 199 | -1.519 | 5.40E-03 |
| PLCD3 | Phospholipase C Δ 3 | -1.514 | 7.22E-03 |
| OTTHUMG00000017601 | OTTHUMG00000017601 | -1.511 | 8.89E-03 |
| INAVA | Innate immunity activator | -1.509 | 1.48E-02 |
| SNORA5A | Small nucleolar RNA, H/ACA box 5A | -1.507 | 2.35E-04 |
| RNA5SP242 | RNA, 5S ribosomal pseudogene 242 | -1.475 | 2.45E-03 |
| ACSL5 | acyl-CoA synthetase long chain family member 5 | -1.462 | 6.89E-03 |
| GPR56 | Adhesion G protein-coupled receptor G1 | -1.460 | 3.64E-02 |
| TRNP1 | TMF1 regulated nuclear protein 1 | -1.457 | 2.31E-02 |
| DNTTIP1 | Deoxynucleotidyltransferase terminal interacting protein 1 | -1.447 | 1.85E-03 |
| SIK2 | Salt inducible kinase 2 | -1.441 | 3.54E-04 |
| RNA5SP202 | RNA, 5S ribosomal pseudogene 202 | -1.429 | 6.70E-03 |
| DCBLD2 | Discoidin, CUB and LCCL domain containing 2 | -1.415 | 9.19E-05 |
| SNORA84 | Small nucleolar RNA, H/ACA box 84 | -1.410 | 1.32E-03 |
| LOC285419 | LOC285419 | -1.403 | 1.38E-03 |
| FRMD4A | FERM domain containing 4A | -1.394 | 7.18E-04 |
| HMBS | Hydroxymethylbilane synthase | -1.385 | 5.77E-04 |
| LOC100287896 | LOC100287896 | -1.382 | 4.01E-02 |
| RNY4 | RNA, Ro60-associated Y4 | -1.378 | 4.37E-02 |
| SNORA28 | [Small nucleolar RNA, H/ACA box 28](https://www.genenames.org/data/gene-symbol-report/#!/hgnc_id/HGNC:32618) | -1.374 | 1.97E-04 |
| VPS35L | VPS35 endosomal protein sorting factor like | -1.370 | 7.10E-03 |
| SNORD8 | Small nucleolar RNA, C/D box 8 | -1.368 | 1.75E-02 |
| HPCAL1 | Hippocalcin like 1 | -1.363 | 1.75E-03 |
| RNA5S17 | NA, 5S ribosomal 17 | -1.355 | 7.18E-03 |
| KLB | Klotho β | -1.354 | 3.09E-02 |
| TLCD1 | TLC domain containing 1 | -1.352 | 5.76E-04 |
| OTUB2 | OTU deubiquitinase, ubiquitin aldehyde binding 2 | -1.328 | 8.29E-03 |
| DHX8 | DEAH-box helicase 8 | -1.327 | 8.45E-03 |
| ADIRF | Adipogenesis regulatory factor | -1.317 | 6.84E-03 |
| IRAK2 | Interleukin 1 receptor-associated kinase 2 | -1.311 | 8.25E-03 |
| BIRC3 | Baculoviral IAP repeat containing 3 | -1.311 | 1.96E-02 |
| NCKAP5 | NCK-associated protein 5 | -1.306 | 6.05E-04 |
| CDC6 | Cell division cycle 6 | -1.304 | 2.99E-02 |
| ICOSLG | inducible T cell costimulator ligand | -1.302 | 2.51E-05 |
| SPRY1 | sprouty RTK signaling antagonist 1 | -1.301 | 7.55E-03 |
| CD69 | CD69 molecule | -1.301 | 1.15E-03 |
| RNA5SP149 | RNA, 5S ribosomal pseudogene 149 | -1.290 | 5.01E-03 |
| IFIH1 | Interferon induced with helicase C domain 1 | -1.287 | 2.61E-02 |
| NFE2L3 | Nuclear factor, erythroid 2 like 3 | -1.285 | 7.10E-03 |
| SNORA21 | Small nucleolar RNA, H/ACA box 21 | -1.281 | 7.66E-05 |
| RNA5SP19 | RNA, 5S ribosomal pseudogene 19 | -1.279 | 5.20E-03 |
| DHX32 | DEAH-box helicase 32 (putative) | -1.278 | 8.57E-03 |
| STMN3 | Stathmin 3 | -1.278 | 1.75E-04 |
| FAM20C | FAM20C golgi associated secretory pathway kinase | -1.273 | 3.33E-02 |
| SCARNA5 | Small Cajal body-specific RNA 5 | -1.271 | 2.92E-03 |
| FAM107B | Family with sequence similarity 107 member B | -1.267 | 4.50E-03 |
| THBS2 | Thrombospondin 2 | -1.264 | 1.94E-03 |
| CACNG6 | Calcium voltage-gated channel auxiliary subunit γ 6 | -1.262 | 2.92E-02 |
| OTTHUMG00000159061 | OTTHUMG00000159061 | -1.256 | 2.59E-02 |
| SNORA76 | Small nucleolar RNA, H/ACA box 50C | -1.252 | 7.89E-04 |
| SH2B3 | SH2B adaptor protein 3 | -1.249 | 4.26E-02 |
| SNORA52 | Small nucleolar RNA, H/ACA box 52 | -1.247 | 1.36E-02 |
| STK39 | Serine/threonine kinase 39 | -1.241 | 1.21E-04 |
| GDA | Guanine deaminase | -1.239 | 4.64E-02 |
| RETREG3 | Reticulophagy regulator family member 3 | -1.238 | 3.27E-03 |
| PSMC3IP | PSMC3 interacting protein | -1.233 | 4.63E-02 |
| RECQL | RecQ like helicase | -1.222 | 1.08E-02 |
| RNU5A-2P | RNA, U5A small nuclear 2, pseudogene | -1.222 | 1.78E-03 |
| OTTHUMG00000152880 | OTTHUMG00000152880 | -1.210 | 2.47E-02 |
| XXYLT1-AS2 | XXYLT1 antisense RNA 2 | -1.202 | 2.67E-02 |
| SCARNA9 | Small Cajal body-specific RNA 9 | -1.200 | 4.09E-03 |
| LIPT2 | Lipoyl(octanoyl) transferase 2 | -1.198 | 3.91E-02 |
| RNU5E-4P | RNA, U5E small nuclear 4, pseudogene | -1.197 | 3.56E-02 |
| OTTHUMG00000165030 | OTTHUMG00000165030 | -1.187 | 2.37E-02 |
| RNA5SP74 | RNA, 5S ribosomal pseudogene 74 | -1.185 | 7.42E-03 |
| CYP2B6 | Cytochrome P450 family 2 subfamily B member 6 | -1.184 | 2.90E-02 |
| RHOF | Ras homolog family member F, filopodia associated | -1.184 | 3.82E-03 |
| AFAP1L2 | Actin filament associated protein 1 like 2 | -1.181 | 2.03E-02 |
| VTRNA1-3 | Vault RNA 1-3 | -1.178 | 3.12E-03 |
| MANSC1 | MANSC domain containing 1 | -1.175 | 3.61E-02 |
| ETV4 | ETS variant transcription factor 4 | -1.175 | 5.36E-03 |
| SNORA31 | Small nucleolar RNA, H/ACA box 31 | -1.173 | 4.17E-02 |
| IL1RAPL1 | Interleukin 1 receptor accessory protein like 1 | -1.173 | 4.66E-02 |
| CDC42EP3 | CDC42 effector protein 3 | -1.172 | 9.55E-05 |
| SNORD61 | Small nucleolar RNA, C/D box 61 | -1.171 | 1.77E-02 |
| RNA5SP191 | RNA, 5S ribosomal pseudogene 191 | -1.168 | 7.68E-03 |
| EPHA2 | EPH receptor A2 | -1.165 | 3.14E-02 |
| TIMM8B | Translocase of inner mitochondrial membrane 8 homolog B | -1.165 | 5.38E-03 |
| THSD4 | Thrombospondin type 1 domain containing 4 | -1.162 | 3.07E-02 |
| FEZ1 | Fasciculation and elongation protein ζ 1 | -1.158 | 1.22E-02 |
| HROB | Homologous recombination factor with OB-fold | -1.157 | 2.24E-02 |
| RNA5SP335 | RNA, 5S ribosomal pseudogene 335 | -1.157 | 3.94E-03 |
| NFKBIE | NFKB inhibitor epsilon | -1.156 | 4.20E-02 |
| MIR4760 | MicroRNA 4760 | -1.147 | 7.69E-03 |
| SNORA80 | Small nucleolar RNA, H/ACA box 80A | -1.146 | 3.34E-02 |
| NT5E | 5'-nucleotidase ecto | -1.146 | 4.11E-03 |
| RNA5SP493 | RNA, 5S ribosomal pseudogene 493 | -1.146 | 1.57E-02 |
| SNORA10 | Small nucleolar RNA, H/ACA box 10 | -1.136 | 4.62E-04 |
| NPAT | Nuclear protein, coactivator of histone transcription | -1.136 | 2.13E-02 |
| RNA5SP150 | RNA, 5S ribosomal pseudogene 150 | -1.135 | 4.08E-03 |
| MLX | MAX dimerization protein MLX | -1.133 | 4.91E-02 |
| PLEC | Plectin | -1.128 | 1.86E-03 |
| B4GALNT3 | β-1,4-N-acetyl-galactosaminyltransferase 3 | -1.128 | 3.45E-02 |
| RAB5C | RAB5C, member RAS oncogene family | -1.123 | 7.40E-03 |
| ABR | ABR activator of RhoGEF and GTPase | -1.121 | 1.37E-02 |
| ARL4C | ADP ribosylation factor like GTPase 4C | -1.117 | 3.46E-02 |
| FSCN1 | Fascin actin-bundling protein 1 | -1.116 | 1.67E-04 |
| IFT46 | Intraflagellar transport 46 | -1.115 | 3.29E-02 |
| HSD11B1 | Hydroxysteroid 11-β dehydrogenase 1 | -1.114 | 3.43E-02 |
| SNORA70D | Small nucleolar RNA, H/ACA box 70D | -1.114 | 5.84E-03 |
| C19orf33 | Chromosome 19 open reading frame 33 | -1.113 | 1.56E-03 |
| SNX19 | Sorting nexin 19 | -1.111 | 4.02E-04 |
| RNA5S9 | RNA, 5S ribosomal 9 | -1.110 | 3.74E-03 |
| ENDOD1 | Endonuclease domain containing 1 | -1.109 | 2.28E-02 |
| CCND1 | Cyclin D1 | -1.108 | 3.92E-02 |
| RNA5SP403 | RNA, 5S ribosomal pseudogene 403 | -1.107 | 6.69E-04 |
| SNORA53 | Small nucleolar RNA, H/ACA box 53 | -1.106 | 1.83E-02 |
| GATA6 | GATA binding protein 6 | -1.100 | 7.49E-03 |
| WNT7B | Wnt family member 7B | -1.100 | 6.03E-03 |
| SNORA79 | Small nucleolar RNA, H/ACA box 79 | -1.099 | 2.61E-02 |
| RNU6-8 | RNA, U6 small nuclear 8 | -1.098 | 8.18E-03 |
| RNU5E-2P | RNA, U5E small nuclear 2, pseudogene | -1.090 | 2.66E-02 |
| RNA5SP263 | RNA, 5S ribosomal pseudogene 263 | -1.087 | 1.92E-03 |
| UBD | Ubiquitin D | -1.085 | 3.36E-02 |
| SCARNA6 | Small Cajal body-specific RNA 6 | -1.071 | 7.25E-03 |
| RNA5SP145 | RNA, 5S ribosomal pseudogene 145 | -1.071 | 1.56E-03 |
| RNA5SP399 | RNA, 5S ribosomal pseudogene 399 | -1.070 | 3.55E-03 |
| PHLDA2 | Pleckstrin homology like domain family A member 2 | -1.067 | 2.04E-02 |
| H2AC21 | H2A clustered histone 21 | -1.067 | 2.32E-02 |
| STX11 | Syntaxin 11 | -1.066 | 3.14E-03 |
| ANKRD20A8P | Ankyrin repeat domain 20 family member A8, pseudogene | -1.064 | 8.59E-03 |
| ZC3H12C | Zinc finger CCCH-type containing 12C | -1.060 | 2.83E-03 |
| RNA5SP298 | RNA, 5S ribosomal pseudogene 298 | -1.059 | 6.97E-03 |
| TNFRSF9 | TNF receptor superfamily member 9 | -1.056 | 1.42E-02 |
| SNORA23 | Small nucleolar RNA, H/ACA box 23 | -1.055 | 1.85E-02 |
| FAM135A | Family with sequence similarity 135 member A | -1.052 | 4.60E-02 |
| OTTHUMG00000132571 | OTTHUMG00000132571 | -1.051 | 1.61E-02 |
| OTTHUMG00000161342 | OTTHUMG00000161342 | -1.048 | 4.27E-03 |
| CNIH3 | Cornichon family AMPA receptor auxiliary protein 3 | -1.042 | 1.20E-04 |
| ANKRD18A | Ankyrin repeat domain 18A | -1.042 | 1.07E-02 |
| RNA5SP290 | RNA, 5S ribosomal pseudogene 290 | -1.040 | 5.41E-03 |
| DDX58 | DExD/H-box helicase 58 | -1.040 | 1.49E-02 |
| SNORD14B | Small nucleolar RNA, C/D box 14B | -1.038 | 2.74E-02 |
| RNU5B-1 | RNA, U5B small nuclear 1 | -1.035 | 4.67E-02 |
| RHOJ | Ras homolog family member J | -1.032 | 1.44E-04 |
| RNA5SP429 | RNA, 5S ribosomal pseudogene 429 | -1.029 | 4.50E-03 |
| ZNF341-AS1 | ZNF341 antisense RNA 1 | -1.027 | 1.34E-02 |
| OTTHUMG00000009734 | OTTHUMG00000009734 | -1.026 | 2.71E-03 |
| LINC02100 | Long intergenic non-protein coding RNA 2100 | -1.026 | 1.22E-02 |
| SCARNA10 | Small Cajal body-specific RNA 10 | -1.026 | 4.03E-03 |
| RNA5SP286 | RNA, 5S ribosomal pseudogene 286 | -1.025 | 1.73E-03 |
| SLCO4A1 | Solute carrier organic anion transporter family member 4A1 | -1.024 | 9.72E-03 |
| RNU2-3P | RNA, U2 small nuclear 3, pseudogene | -1.019 | 2.80E-03 |
| RGS2 | Regulator of G protein signaling 2 | -1.019 | 1.02E-02 |
| NQO1 | NAD(P)H quinone dehydrogenase 1 | -1.019 | 1.61E-02 |
| SFR1 | SWI5 dependent homologous recombination repair protein 1 | -1.018 | 6.95E-03 |
| RPL27 | Ribosomal protein L27 | -1.016 | 1.44E-02 |
| RNA5SP440 | RNA, 5S ribosomal pseudogene 440 | -1.016 | 6.63E-03 |
| MALL | Mal, T cell differentiation protein like | -1.015 | 2.47E-02 |
| EI24 | EI24 autophagy-associated transmembrane protein | -1.012 | 2.65E-05 |
| RNA5SP86 | RNA, 5S ribosomal pseudogene 86 | -1.011 | 9.87E-03 |
| MGAT4B | α-1,3-mannosyl-glycoprotein 4-β-N-acetylglucosaminyltransferase B | -1.009 | 1.75E-02 |
| BUD13 | BUD13 homolog | -1.005 | 6.38E-03 |
| SNORA49 | Small nucleolar RNA, H/ACA box 49 | -1.002 | 2.89E-02 |

*^¶^Genes differentially expressed in SUIT-2 PDAC tumor cells exposed to long-term extracellular acidity (L.A. group) were determined by Affymetrix GeneChip Human Transcriptome Array 2.0, and compared with gene expression profiles of cells treated with acute short-term acidification (S.A. group). A total of 220 genes (Log_2_FC ≤ -1, p-value < 0.05) were identified to be significantly down-regulated in the L.A. cell group as compared to those in the S.A. cell group. The very few genes up- or down-regulated found in SUIT-2 tumor cells exposed to HEPES/PIPES buffer were excluded for the purpose of paired analyses (see comparison between Ctrl and Buff cell groups).*
